# Lung retention, distribution and persistence of polymer particles in rats exposed via inhalation

**DOI:** 10.1186/s12989-025-00655-4

**Published:** 2026-01-17

**Authors:** Emanoela Thá, Lan Ma-Hock, Markus Rueckel, Till Gruendling, Wendel Wohlleben, Bernd Reck, Robert Landsiedel

**Affiliations:** 1 Experimental Toxicology and Ecology, BASF SE, Ludwigshafen am Rhein, Germany; 2 Material Science, BASF SE, Ludwigshafen am Rhein, Germany; 3 Analytical Science, BASF SE, Ludwigshafen am Rhein, Germany; 4 Pharmacy, Pharmacology and Toxicology, Freie Universität Berlin, Berlin, Germany

**Keywords:** Polymer, Nanoplastics, Lung, Lymph node, In vivo inhalation study, Internal dose, Pyrolysis-GC/MS

## Abstract

**Background:**

Microplastics have been repeatedly detected in the human body, yet uncertainties surround their bioavailability and fate due to experimental challenges and limitations, especially regarding their nano-sized counterparts. Knowing that toxicokinetics information is essential for accurate risk assessment and management, this research aimed to (1) evaluate different sample preparation and quantification methods for nanoplastics particles in mammalian tissue, and (2) investigate the lung retention, bioavailability and fate of these particles.

**Methods:**

In this study, rats inhaled aerosols with up to 50 mg/m^3^ of Nile Red-labeled polystyrene (PS-NR) or unlabeled polyamide particles (PA-6) particles for 28 days. The tissues were analyzed for the presence of polymer particles. PS-NR were quantified in formalin-fixed tissue by confocal fluorescence laser microscopy with semi-automatic imaging analysis, and PA-6 particles were quantified in dried tissues by pyrolysis-gas chromatography-mass spectrometry (Py-GC/MS).

**Results:**

PA-6 deposition was detected and quantified in lung and lymph nodes. Deposition of PS-NR was quantified in lungs and lung-draining lymph nodes, but no particles were detected in the liver, spleen, and kidneys. The lung burdens and translocation to the draining lymph nodes were similar for both particles, and particles were still detectable after the end of the exposure periods (five weeks for PS-NR and 13 weeks for PA-6).

**Conclusions:**

This work highlights limitations and applicability of the various methods for sample preparation, detecting and quantifying polymer particles in mammalian tissues. In addition, it provides reliable data on the internal dose of inhaled polymer particles.

**Graphical Abstract:**

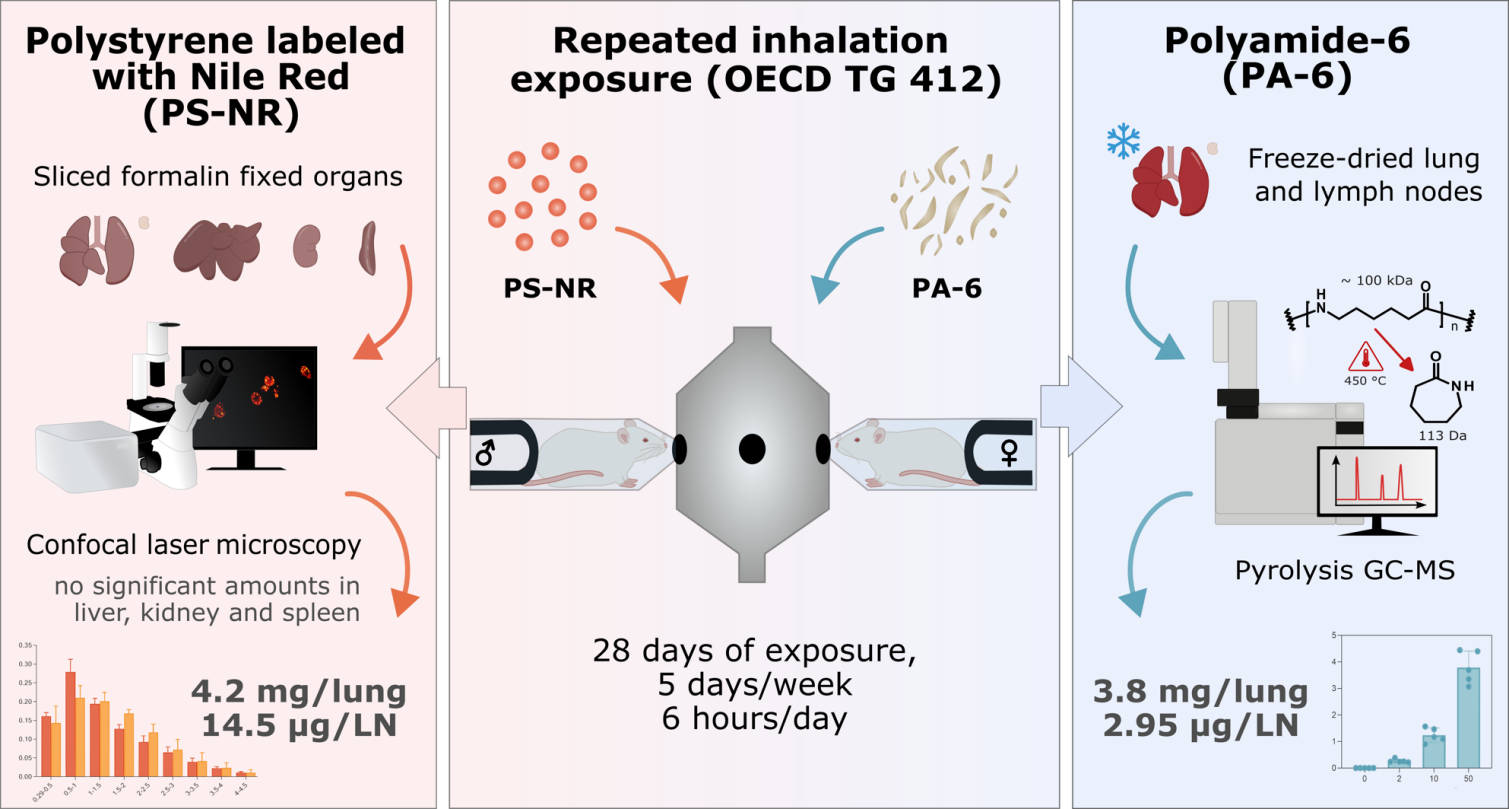

**Supplementary Information:**

The online version contains supplementary material available at 10.1186/s12989-025-00655-4.

## Background

Polymers are highly indispensable materials in numerous industry sectors such as packaging, automotive, textile, and construction; thus, being ubiquitous in daily life [1–4]. Microplastics and nanoplastics are solid polymer particles that are receiving a significant amount of attention regarding their potential health effects, bioaccumulation, and persistence in the environment [5, 6]. These particles can be either manufactured as primary nano- or microplastics, or formed by degradation and weathering of plastics (incidental or secondary nano- and microplastics) [7, 8]. Although upper and lower size limits of each category are not well defined, and a unified terminology is not currently available, it is generally well accepted that microplastics have a diameter of less than 5 mm, and nanoplastics have less than 1000 nm [9]. This classification will be used throughout this paper.

Due to their widespread presence in all environmental compartments (water, soil, air), humans are exposed to microplastics through various routes [7]. Inhalation is estimated to contribute to approximately half of the annual exposure to microplastics [10], with sources including spray applications [11], 3D-printing [11], wear and tear of textiles [7, 12], and outdoor air [13–15].

The bioavailability, distribution and persistence in the body (i.e., toxicokinetics) of these materials is not yet well examined. While multiple studies have claimed identification of microplastics in human samples, such as lung [16], blood [17], placenta [18, 19], carotid artery plaque [20], the available evidence can be compromised by experimental limitations, including contamination of samples with environmental plastics and limits of detection [21–23]. The uncertainty increases for nano-sized plastics, as their detection using chemically-selective microscopic techniques is currently not feasible and advancements in technologies for their detection are needed [21, 24].

For the respiratory tract, the particle size determines the region of the airways where the particle deposits and how they can be absorbed and become systemically available [25, 26]. Conventionally, particles larger than 4 μm are considered to primarily deposit in the tracheobronchial regions of humans and are cleared via the mucociliary escalator, whereas particles smaller than 4 μm are deemed to be able to reach the deep lung [27]. Particles that reach the alveolar region could cross the air-blood barrier. However, the behavior of poorly soluble particles in the respiratory tract can vary based on the particle size and physicochemical properties [28], and the understanding of the behavior of nanoplastics is limited.

Adding to the challenge, there is a lack of standardized methodology for the reliable identification, quantification, and characterization of microplastics in biological matrices [29–32]. Often, biological samples are dissolved by alkaline or enzymatic treatments and microplastics are subsequently quantified by analytical methods such as micro(µ)-Raman spectrometry [29, 31, 32]. However, the matrix is often incompletely removed and the digestion method can have an impact in the polymer, which may hinder the accurate quantification [30, 33].

The available knowledge regarding the toxicokinetics of microplastics in mammals, particularly when considering inhalation as the route of exposure, remains limited and subject to controversy. In addition, according to De Boever et al. [21], almost 80% of studies using microplastics have employed polystyrene model particles, mainly due to their low cost and easy production. Nonetheless, their limited resemblance to plastic particles commonly found in the environment raises concerns about their applicability in toxicity assessments. It is essential to explore alternative microplastic materials that may offer more environmentally relevant insights into their toxicity. One of the most abundant microplastic in the indoor air is polyamide (i.e., nylon) [34–36], and it has been detected in samples of human respiratory tract [37, 38]; thus, showing high environmental relevance regarding inhalation exposure [34–36]. Therefore, in our study, Wistar rats were exposed via inhalation to two different nanoplastic particles, an engineered polystyrene particle labeled with Nile Red (PS-NR) or unlabeled polyamide-6 (PA-6). Different analytical approaches were explored to detect and quantify PS-NR and PA-6 in the lung and extrapulmonary organs, aiming to [1] address the current gap in nanoplastic quantification within biological tissues and [2] provide insights into lung burden, particle distribution across other tissues, and their potential persistence in the body. To provide the analytical work with the level of detail it deserves, this paper focuses solely on methods for investigating organ and tissue distribution, and the biological effects will be reported in separate papers.

## Materials and methods

### Test material: polystyrene

Polystyrene (PS) particles were synthesized and labeled *in house.* The suitability of two labeling approaches was investigated prior to the study (see SI for details): [1] covalent incorporation by using a Nile Blue derivative with polymerizable double bond as comonomer in the emulsion polymerization of styrene; [2] Nile Red incorporation into pre-formed PS particles via van-der-Waals interactions using a solvent swelling approach. Due to the considerably higher fluorescence intensity, the particles labeled using the swelling method were chosen for the further studies described in this paper.

### Synthesis

Unlabeled PS particles were synthesized by semi-batch emulsion polymerization of styrene using 6 parts per hundred monomer (pphm) sodium dodecyl sulfate (SDS) as surfactant and 0.5 pphm sodium peroxodisulfate (NaPS) as initiator. In a 2 L glass polymerization reactor equipped with an anchor stirrer (150 rpm), a precharge containing 300 g of de-ionized water, 7.5 g of a 15 wt% aqueous solution of SDS and 7.5 g of sodium bicarbonate was heated to 85 °C and purged with nitrogen. In a feed tank, an emulsion was prepared from 300 g styrene, 50 g of a 15 wt% solution of SDS and 310 g water. Twenty grams of the styrene emulsion and 6.5 g of a 7 wt% aqueous solution of NaPS were rapidly added to the preheated precharge in the reactor. After a waiting time of 10 min, the remaining styrene emulsion was fed to the reactor in 3 h. At the same time, 60 g of a 7 wt% solution of NaPS was dosed to the reactor in 3 h 15 min. After completion of the NaPS feed, 60 g of a 15 wt% solution of SDS was added to the reactor and the latex was stirred for another 30 min to complete the polymerization. To reduce the residual styrene monomer, 4.5 g of a 10 wt% aqueous solution of tert-butyl hydroperoxide and 5.7 g of a 13 wt% aqueous solution sodium acetone bisulfite was dosed in parallel to stirred latex at 85 °C over the course of 1 h. After cooling to room temperature, a polystyrene latex with a solid content of 29.5% and a pH of 2.6 was obtained. A particle size of 100 nm with a polydispersity of 0.062 was measured by dynamic light scattering.

#### Nile red labeling

Nile Red was incorporated by solvent swelling by a procedure that was optimized from previous literature [39] (Figure S1). Specifically, 67.7 g of the PS suspension were diluted to 400 g by MilliQ water, resulting in 2% PS concentration. Independently, 90 mg Nile Red (CAS-No: 7385-67-3, cat. no. 72485, Merck, Darmstadt, Germany) was dissolved in 30 mL tetrahydrofuran (THF). In several parallel batches of each 180 g of the diluted PS suspension, 30 mL of the THF containing Nile Red was added dropwise under stirring. Then, 240 g MilliQ water were added, and homogenized by stirring for 15 min.

#### Washing

The batch was split into 12 ultracentrifuge polypropylene vials (prod. no. 326823, Beckman Coulter, Brea, USA), and particles were pelleted at 25 °C for 6 h at 15,000 rpm (Optima XL-80 K, Beckman Coulter, Brea, USA). The supernatant, containing free surfactant and free Nile Red, was discarded. 10% of the volume was retained. The volume was restored to two thirds by MilliQ water, and the pellet was resuspended by repeated intake via a disposable pipette. Then, the volume was restored fully by MilliQ water and pooled. The pooled suspension was redispersed using a vortex mixer for 1 min, followed by 5 min in a sonication bath, and then redispersed by vortex mixing for an additional 1 min The washing procedure was performed three times, resulting in a volumetric dilution of the non-particulate components (THF, free Nile Red, free surfactant, oligomers) by a factor (10%)^3^ = 0.001. One complete pooled suspension was generated.

The parameters of the washing procedure were optimized to balance high staining efficiency against low agglomeration (induced by swelling with THF and hard pelleting). Despite the efforts to redisperse, a small content of agglomerates needed to be removed by a softer centrifugation at 3000 rpm for 20 min in a conical 50 mL centrifuge tube (prod. no.: 339652, Thermo Fisher Scientific, Waltham, USA). At this speed, the sedimentation velocity of PS nanoplastics was so slow that losses were minor, but agglomerates were removed effectively. In this approach, the supernatant was kept and pooled, whereas the sediment was discarded.

#### Adjustment of properties

To minimize secondary effects, the pH was adjusted to pH 7 with NaOH. To ensure storage stability, the lost surfactant was compensated by addition of 0.014% of SDS. This concentration is minimum to ensure stability, and is orders of magnitude lower than the typical side components in commercial “PS beads” or “NIST-traceable calibration latices” [40]. Additionally, a mixture effect by biocide or oligomer can be excluded [41].

#### Characterization of intrinsic properties

The solid content was determined gravimetrically as 1.42 wt%. The final pooled suspension had a volume of 8.4 L, containing thus a total of 119 g of labeled and washed PS nanoplastic. The remaining content of THF was determined at 200 ppm by gas chromatography with flame-ionization detection (GC-FID). Free Nile Red was determined at 0.55 mg/L by UV/Vis spectrometry of a particle-free filtrate. The size distribution was determined by Analytical Ultracentrifugation (AUC) according to the JRC Method Manual [42]. The size distribution had low polydispersity, resulting in close similarity of size distributions in mass metrics and in number metrics (Table 1, Figure S2A).

**Table 1:** PS-NR size distribution in mass and number metrics determined by analytical ultracentrifugation

	Size distribution in mass metrics [nm]	Size distribution in number metrics [nm]
d10	68	58
d50	89	78
d90	116	101

### Test material: polyamide

Commercial PA-6 plastic granules were converted by solvent precipitation into nanoplastics. The original PA-6 was a grade with intended use in carpets or in compounding. Both uses may be linked to human inhalation exposure. It was specified as containing no additives. The synthesis and characterization of nano-sized PA-6 (designated there as “PA-6_precip”) are described in detail in Santizo et al. [43]. In short, polymer was dissolved in diethylene glycol (DEG), corresponding to a concentration of 3% w/w. The heated PA-6/DEG solution (240 °C) was then added within 1 h to room temperature water containing polyvinyl alcohol (PVA) as dispersant, inducing precipitation in the form of a nanoplastics slurry. The PA-6 slurry was filtered (14 μm), then washed in total three times by different steps of centrifugation and resuspension to reduce the content of DEG and PVA from the suspension. The supernatant from each centrifuge cycle was removed and discarded. In the final suspension, 0.5% DEG remained. An IR-spectral match between the precipitated material and PA-6 granulates confirmed particle identity. A shoulder at 1730 cm^-1^ illustrated the presence of a minor component of the PVA, which was quantified at a content of 0.14 wt% via field-flow fractionation. All measured properties indicated that the PA-6_precip is representative of commercial PA-6, including the molar mass distribution, the crystallization peak and melting point, the molecular mobility and the absence of charge. With the given particle size (Table 2, Figure S2B) and density, the final mass concentration of 39 g PA-6 per kg suspension represents a particle number concentration of 6.2 × 10^19^ /L and consists entirely of respirable nanoplastics.

**Table 2:** PA-6 size distribution in mass and number metrics determined by analytical ultracentrifugation

	Size distribution in mass metrics [nm]	Size distribution in number metrics [nm]
d10	34.5	29.9
d50	66.7	37.4
d90	213.2	55.5

### Screening of the stability against leaching and dissolution

To investigate whether Nile Red could leach to non-polar compartments of the tissue due to the use of THF in the swelling method, the labeled particles were embedded in a nonpolar acrylate matrix (Acronal 3659X) in a ratio of 1:1000. The dispersion was quickly dried at 70 °C and the fluorescence was measured immediately after the drying and after five days at 23 °C.

Here, we additionally investigate stability against dissolution, and potential transformation, in physiological simulant media. A continuous flow system (CFS) was used as described by ISO/TR 19057:2017 [44], and as previously used for organic nanomaterials [45, 46], where the particles are held between ultrafiltration 3 kDa membranes (corresponding to a 1 nm cutoff) at 37 °C, and are exposed to a continuous 2 mL/hour flow of the simulant fluid. We tested independently in lung lining fluid (Gamble’s, pH 7.4) [44, 47] and in phagolysosomal simulant fluid (PSF, pH 4.5) [44, 48]. After 24 h, the remaining particles are re-analyzed by transmission electron microscopy (TEM) according to the JRC Methods Manual [42], with *n* > 1000 particles evaluated for their size and aspect ratio.

To investigate potential leaching of plastics-associated chemicals, the eluted simulant fluids of PS-NR and PA-6 were analyzed by gas chromatography-mass spectrometry (GC/MS) for the monomers styrene and caprolactam, respectively, as described by Santizo et al. [43].

### Pilot studies for extraction of polymers from tissue

Several sample preparation techniques were evaluated by spiking the tested particles (PS-NR and PA-6) to organs of untreated animals. The techniques include the use of THF as an extraction solvent, chemical digestion with a basic piranha solution (a 3:1 mixture of ammonium hydroxide (NH_4_OH) and hydrogen peroxide (H_2_O_2_)) and Soluene^®^ 350, as well as enzymatic digestion using a protease in Tris-buffer. However, these methods did not yield satisfactory results due to poor recovery, either resulting from the destruction of the polymers, leaching of the fluorescence tag, or the formation of unwanted foam.

For PA-6, a protocol involving dispersion and extraction from the dried tissues in 1,1,1,3,3,3-Hexafluoro-2-propanol (HFIP) was successfully established for quantifying PA-6 in both lung and lymph nodes (LNs) tissues (details in Sect. "Tissue preparation for pyrolysis GC-MS"). However, when the same protocol was tested in the liver, matrix contamination was excessive, and the required limits of detection were not achieved.

Therefore, a sample enrichment protocol based on solid phase extraction (SPE) was attempted for PA-6. Unfortunately, a significant background contamination of PA-6 was observed in three out of 11 tested SPE cartridges with different types of solid phase material and from different manufacturers. Further attempts at method development were unsuccessful, as the chosen conditions eluted either both the matrix and polymer or none of each. Therefore, PA-6 could only be quantified in lung and LNs.

Finally, the chosen techniques for sample preparation were the manual tissue sectioning for confocal laser microscope analysis for PS-NR (described in Sect. "Blood smear and tissue sectioning for confocal laser microscopy analysis") and HFIP extraction for pyrolysis-GC/MS (Py-GC/MS) for PA-6 NR (described in Sect. "Tissue preparation for pyrolysis GC-MS").

### In vivo study

#### Animals

Male (PS-NR) and female (PA-6) Wistar rats (Crl: WI(Han)), 9 weeks old and healthy, were obtained from Charles River Laboratories (Germany) and kept in groups (five animals/cage in polysulfonate cages (2065 cm^3^) with dust-free bedding. Enrichment was provided with wooden gnawing blocks and play tunnels. The animal accommodations were maintained at 22 ± 2 °C with a relative humidity of 55 ± 10% and a 12-hour light/dark cycle. The rats had ad libitum access to certified feed and water, except during treatment. Before exposure, the rats were allowed to acclimatize to the housing for approximately two weeks, followed by a three-day acclimatization period to the inhalation apparatus.

Animals of different sexes were used to align with animal welfare considerations, and the specific study objectives related to toxicological examinations, which will be reported in a separate manuscript. For the purpose of this paper, the use of different sexes does not influence the interpretation or outcomes of the analytical results.

#### Study design

The study was designed according to OECD Test Guideline 412 [50]. Test concentrations were selected in accordance with the guideline, and intentionally covered a range exceeding real-life exposure scenarios. This approach allows the identification of concentrations causing observable effects and resulting in organ burdens above the limits of detection of the used methods. Animals were randomized based on weight into three separate groups: the main group (MG) and two recovery groups (post-exposure observation group 1 (PEG1) and post-exposure observation group 2 (PEG2)). The rats were nose-only exposed to PS-NR (5 and 50 mg/m^3^) or PA-6 (2, 10 and 50 mg/m^3^) while being restrained in glass tubes attached to the inhalation chamber. The exposures were performed for 6 h/day, 5 days/week for 28 days. A concurrent control group was exposed to water. One day after the last exposure (MG), as well as about five (PEG1) and 13 weeks (PEG2) post-exposure, the animals were sacrificed. Tissues were examined for polymers. The test groups are summarized in Table 3 and detailed in Table S1.

**Table 3:** Test groups and corresponding exposed atmospheric concentrations and post exposure period prior to sacrifice

	PS-NR		PA-6	
	Concentrations [mg/m^3^]	Post exposure period [weeks]	Concentrations [mg/m^3^]	Post exposure period [weeks]
Main group (MG)	0, 5 and 50	0	0, 2, 10 and 50	0
Post-exposure observation group 1 (PEG1)	0 and 50	5	0 and 50	5
Post-exposure observation group 2 (PEG2)	0 and 50	13	0 and 50	13

#### Aerosol generation and inhalation

To generate the aerosol, a particle suspension (MilliQ water in the control group) was supplied to 2-component atomizers (Model 970, Schlick, Untersiemau, Germany) using piston metering pumps (Duratec, Hockenheim, Germany). Compressed air was used to spray the suspension into the inhalation system. The target concentrations were achieved by adjusting the pump rate while diluting the aerosol with conditioned fresh air.

The inhalation chamber, a custom-made 90-L cylindrical stainless-steel chamber, operated at a positive pressure to prevent dilution of the test material. Rats were restrained in glass tubes with their snouts inside the chamber to ensure exclusive inhalation exposure. The system maintained a daily mean relative humidity of 54% and a temperature of 21.5 °C. To maintain the chamber temperature in desired range, the conditioned air was warmed with circulation thermostats.

#### Aerosol analysis

Aerosol analysis was performed as previously detailed by Ma-Hock et al. [51]. Samples were collected from the breathing zone of the rats using an air sampling station (Millipore, Schwalbach, Germany) at a flow rate of 3 L/min. Aerosol particle concentrations were determined twice daily by sampling on 4.7 cm MN 85/90 BF filters (Macherey-Nagel, Dueren, Germany) and subsequent gravimetric analysis. The sampling time was 120 and 40 min for the low and high aerosol concentrations, respectively. Additionally, real-time monitoring was conducted using a scattered light photometer (VisGuard, Sigrist, Switzerland) throughout the daily exposure period. Cascade impactor measurements were carried out with an 8-stage Marple Personal Cascade Impactor (Sierra-Andersen, Atlanta, Georgia) utilizing sample volumes of 1080 and 360 L for the low and high aerosol concentrations, respectively. The effective aerodynamic cutoff diameters were 21, 15, 10, 6.5, 3.5, 1, 0.7, and 0.4 μm. In the sub-micrometer range, particle size distribution was assessed using a scanning mobility particle sizer (SMPS, Grimm Aerosol Technik, Ainring, Germany). Particle count concentrations in the size range from 0.011 to 1.083 μm were measured (approximately 10 replicates per determination).

#### Blood smear and tissue sectioning for confocal laser microscopy analysis

Before euthanasia, intracardial EDTA-blood samples were withdrawn from anesthetized animals (ketamine (150 mg/kg body weight (b.w.)) / xylazine (15 mg/kg b.w.)) previously exposed to PS-NR. Fifty microliters of EDTA-blood were pipetted onto glass slides and centrifuged in an OMRON Centrifugal Spinner (HEG-SP, Kyoto, Japan). The slides were air-dried, fixed in methanol and stored at room temperature.

Following the intracardiac puncture, the animals were sacrificed by exsanguination from the abdominal aorta and vena cava. Left lung, lung-draining LNs, liver, spleen, and kidneys were fixed in formalin 10% for at least 48 h before processing. The tissues were manually cut into slices with thickness ranging from 200 to 300 μm and width of maximum 0.5 cm. The slices were stored in formalin 10% at room temperature and in the dark until analysis.

#### Tissue preparation for pyrolysis GC-MS

Animals exposed to PA-6 were sacrificed under ketamine (150 mg/kg b.w.) / xylazine (15 mg/kg b.w.) anesthesia by exsanguination from the abdominal aorta and vena cava. Freshly dissected left lung and lung-draining LNs were stored frozen at – 20 °C. The organs were dried at 60 °C for 48 h and the lungs subsequently cut into pieces approximately 1–2 mm in size. The dried lung-pieces and LNs were dispersed in 20 mL and 3 mL HFIP (≥ 99%, Tokyo Chemical Industry, Tokyo, Japan) respectively, by use of an ULTRA-TURRAX® homogenizer (IKA-Werke, Staufen im Breisgau, Germany). The resulting dispersion was filtered through a syringe filter (Minisart RC 20 μm, Sartorius, Göttingen, Germany). Twenty to fifty microliters of the resulting clear filtrate were transferred into pyrolysis crucibles (Frontier Laboratories, Koriyama, Japan) and the solvent was evaporated at 60 °C during approximately 10 min before measurement according to the protocol outlined in "Pyrolysis-GC-MS".

### Detection and quantification of nanoplastic particles

#### Polystyrene

##### Confocal laser microscopy imaging

The presence of PS-NR was investigated in the lungs, lung-draining LNs, kidney, spleen, liver and smeared blood samples of animals exposed to 50 mg/m^3^ PS-NR in the MG and PEG1. To image the distribution of the fluorescently labeled nanoparticles, at least 10 image stacks per tissue slice with arbitrarily chosen positions were taken using SP8 confocal laser-scanning microscope (Leica Microsystems, Wetzlar, Germany) in a semi-automated process. To this end, tissue slices (two slices per sample) were immersed in formalin in a Lab-Tek chamber (Nunc™ Lab-Tek™ II Chamber Slide™ System, prod. no. 155409, Thermo Fisher Scientific, Waltham, USA) and a stainless-steel nut on a piece of foil was used to maintain the slices close to the bottom of the chamber. One blood smear slide per animal from the MG (*n* = 3) was also analyzed. An oil immersion objective lens (HC PL APO CS2, 63x/1.40, Leica Microsystems, Wetzlar, Germany) was employed to image the tissue slices to conduct the imaging process overnight without evaporation of the immersion media.

The scattering contrast provided information about tissue structure and assisted in identifying structures presumed to be macrophages (excitation at 458 nm / emission at 452–465 nm), while the fluorescence contrast allowed visualizing the Nile Red-labeled nanoparticles (excitation at 561 nm / emission at 571–795 nm).

The imaging setup included lateral image dimensions of 246 μm x 246 μm, with a corresponding lateral voxel size of 120 nm. The axial voxel size was set at 300 nm, and the image stacks covered an axial dimension of approximately 100 μm for both lung and LN tissues. The axial dimension was adjusted slightly until the scattering contrast of the tissue became too weak to identify tissue structures. To enhance the sensitivity of the analysis for liver, spleen, kidney and smeared blood samples, the axial size was reduced to 150 nm and frame averaging (4x) was employed.

For an overview of the fluorescence distribution of an entire lung section (length of approximately 0.5 cm), an air-objective lens (5x, 0.15) was used to take four image stacks with a depth of 300 μm using tile scan.

To validate the sensitivity of our imaging process, PS-NR particles were dispersed in an acrylate dispersion (Acronal 3659X) in a ratio of 1:500. The mixture was then coated onto a cover glass in a 30 μm thickness, and quickly dried on a hotplate at 90 °C. The dried uniform acrylate film was imaged via xyz-scans to identify the size distribution of the native, non-sprayed fluorescent nanoparticles.

##### Image processing

Each image stack was processed automatically by an ImageJ Macro using three-dimensional (3D) segmentation of the Nile Red fluorescence contrast based on the plugin Labkit, an automatic segmentation tool with a user-trained pixel classifier [52]. The classifier was trained to discriminate weak objects from the background and ensure clear separation from neighboring objects. A second classifier was trained to be more sensitive but less separative, specifically for analyzing fluorescent objects in remote organs. For that, the sensitivity was increased through frame averaging (4x) and tuning the pixel classifier of the segmentation process to detect even weaker objects at the expense of discriminating adjacent objects. For each identified object, the average intensity and equivalent sphere diameter were calculated, excluding those at the edges of the image stack, while the deepest location of an identified object was also determined to calculate the concentrations of nanoparticles in all scanned tissue volumes.

Due to the limited spatial resolution of the 1.40 NA objective (approximately 200 nm laterally and 500 nm axially), single 89 nm nanoparticles were imaged larger than their real diameter. In the validation test using the embedding acrylate film, single nanoparticles appeared at least 6 voxels (120 nm laterally and 300 nm axially), which reflects an average equivalent sphere diameter of 0.29 μm. Identified objects smaller or equal to 5 voxels were excluded from further analysis and were considered as background noise (Figure S3). Two or more individual nanoparticles located within a distance smaller than the spatial resolution could not be resolved as separate objects, thus being detected as a single larger object.

For the calculation of the detected mass of nanoparticle agglomerates, it was assumed that nanoparticles agglomerate irregularly with a packing density of about 64% [53]. The density of polystyrene was considered 1.05 g/cm^3^, as determined using Helium Pycnometry [43].

#### Polyamide

##### Pyrolysis-GC-MS

The lungs and the lung-draining LNs of animals exposed to 2, 10 and 50 mg/m^3^ PA-6 in the MG, and on animals exposed to 50 mg/m^3^ PA-6 in the PEG1 and PEG2 were analyzed for PA-6. Measurements were performed on an analytical pyrolysis system (EGA/PY-3030D, Frontier Laboratories, Koriyama, Japan) equipped with an autosampler (AS-1020E, Frontier Laboratories, Koriyama, Japan) and coupled to a gas chromatograph (7890B, Agilent, Santa Clara, USA) with mass spectrometric detector (5977 A, Agilent, Santa Clara, USA). Agilent MassHunter Data Acquisition and ChemStation Data Analysis were employed for instrument control and data analysis. Microbalances (MC 5 and MCA225S, Sartorius, Göttingen, Germany) were used for accurate weighing.

The prepared crucibles (see 2.6.6) were flash-pyrolyzed at 450 °C under helium with a transfer-line temperature of 320 °C. The GC injector temperature was 320 °C, with a split of 1:20, a column flow of 0.9 mL/min (Helium) and a septum purge of 3 mL/min. The pyrolysis products were separated on a capillary column (Zebron ZB-50, 30 m x 0.25 mm x 0.5 μm, Phenomenex, Torrance, USA). The temperature program was 40 °C for 1 min, 15 °C/min to 320 °C and a holding time of 7 min at 320 °C. MS transfer-line temperature was 300 °C and measurement was performed in EI-mode (A-tune). The main degradation product of PA-6 (caprolactam) was used as marker fragment (ret. time: 11.5 min, m/z: 113 (quantifier), 30, 55, 84 (qualifier)). Appropriate blanks were run to ascertain the solvent and sampling equipment was free of relevant contaminations.

Stock solutions of the reference were prepared by weighing approximately 49 mg and 62 mg of PA-6 to an accuracy of 0.01 mg into a 25 mL volumetric flask, dissolving the polymer and filling to the mark with 1,1,1,3,3,3-Hexafluoro-2-propanol (HFIP, ≥ 99%, Tokyo Chemical Industry, Tokyo, Japan). Fifty microliters of stock solution were further diluted in 10 mL HFIP and final calibration samples were prepared by pipetting 3 to 300 µL (= 37 to 3700 ng PA-6) for the lungs and 2 to 115 µL (= 20 to 1140 ng PA-6) for the LNs of the diluted stock solutions into a pyrolysis crucible. The solvent was then evaporated at 60 °C for approximately ten minutes prior to injection into the pyrolysis system. Concentrations were determined as total mass of PA-6 per wet organ and subsequently divided by wet organ mass.

The extracts of three selected LN samples were spiked in duplicate each at masses corresponding to 50 and 100 µg PA-6 per LN with recoveries ranging from 63 to 101%. In addition, six blank LN samples were spiked in triplicate each at 5 and 100 µg PA-6 per LN before extraction with recoveries ranging from 101 to 118% (one outlier at 156%).

The extracts of three selected lung samples were spiked in duplicate each at masses corresponding to 40 to 1200 µg PA-6 per lung with recoveries ranging from 97 to 113%.

### MPPD model

The computational Multiple-Path Particle Dosimetry (MPPD) model (software version 3.04, www.ara.com/mppd) was used to estimate the pulmonary deposition fraction of the nanoplastic particles using the parameters described in Table 4.

**Table 4:** Parameters in MPPD for calculating pulmonary deposition and clearance

		PS-NR	PA-6
Airway morphometry	Species	Rat	
	Model	Asymmetric Sprague Dawley	
	Weight (g)	270	200
Inhalant properties	Density (g/cm^3^*)*	1	1
	MMAD (µm)	1.32	1.51
	GSD (µm)	2	2
	Inhalability adjustment	ON	
Exposure condition	Constant exposure		
Exposure scenario	Aerosol concentration (mg/m^3^*)*	50	
	Breathing scenario	Nose only exposure	
Deposition/clearance	Deposition only		

Parameters not described in the table were used as default

### Retained mass and retention half-time calculation

The following equation [54] was used to calculate the total applied mass:

$$\:total\:applied\:mass\:\left[mg\right]=\frac{\begin{array}{c}exposure\:time\:\left[min\right]\times\:lung\:rate\:\left[L\:{min}^{-1}\right]\times\:\\\:aerosol\:concentration\:\left[mg\:{m}^{-3}\right]\end{array}}{1000}$$

According to Snipes [55], the lung ventilation rate for rats is 0.8 L/min/kg b.w. Given that the average body weight of male rats (PS-NR) was approximately 270 g and that of female rats (PA-6) was about 200 g, the corresponding lung ventilation rates were determined as 0.22 L/min and 0.16 L/min, respectively. The percentage of the retained mass in the lungs was calculated relative to the total applied mass.

The clearance constant (*λ*) was calculated using the expression:

$$N(t) = {N_0} \times {{\rm{e}}^{ - \lambda t}}$$

where *N*(*t*) was the lung burden at time point *t*, *N*_0_ was the lung burden shortly after last exposure, with *t* as days after last exposure [56]. The half-time of lung retention was then calculated using the following expression:

$$\:\mathrm{t}1/2 = \frac{ln\:\left(2\right)\:}{\lambda\:}$$

## Results

### Particle stability

**Fig. 1:**
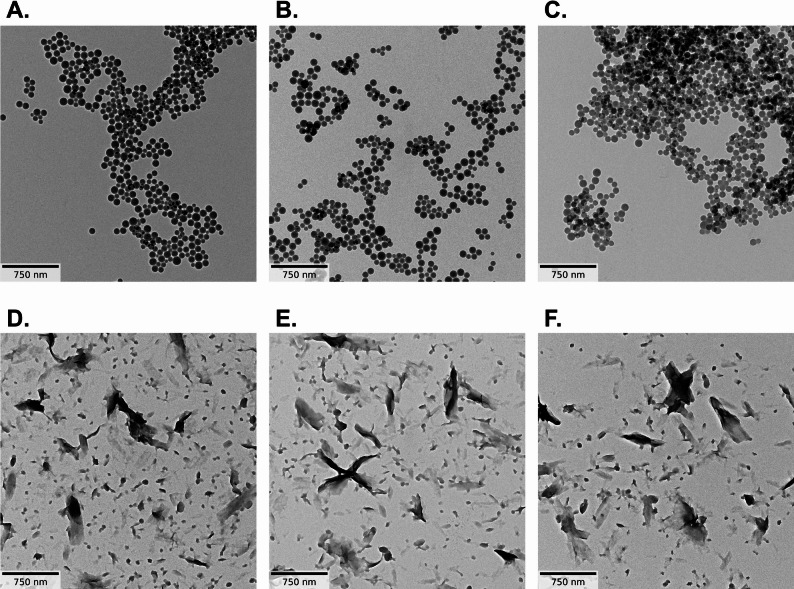
Testing of stability against dissolution or transformation by continuous flow system with TEM evaluation. **A** to **C** shows PS-NR; **D** to **F** shows PA-6. **A** and **D** control in H2O; **B** and **E** after incubation in lung lining fluid (Gamble’s, pH 7.4); **C** and **F** after incubation in phagolysosomal simulant fluid (PSF, pH 4.5). Average and median particle size, and median aspect ratio of the particles (*n* > 1000) obtained with automated image analysis are shown in Table S2

PS is not water-soluble and neither is PA (even though it is hygroscopic and absorbs water) [57, 58]. A priori, the behavior in physiological fluids might be different than in water, and some transformation of particle shape or leaching of plastics-associated chemicals might occur even at low solubility. But neither polymer showed signs of degradation in size or shape in the lung lining simulant fluid (Gamble’s, pH 7.4) [44, 47] or phagolysosomal simulant fluid (PSF, pH 4.5) [44, 48] fluids (Fig. 1), suggesting that both particles are insoluble in biological fluids and would remain stable at their original size and shape following inhalation. The median number metrics size of PA-6 was 49.0 nm in the control, and 49.1 and 50.0 nm in Gamble’s or PSF, respectively. Considering the polydispersity of 40 nm, this change is not significant. The average aspect ratio was 1.5 in the control, and 1.6 after incubation in either fluid (Table S2). The median number metrics size of PS-NR was 75.7 nm in the control, 76.0 nm after Gamble’s, and 75.2 nm after PSF. This change is also not significant, considering the polydispersity of 16 nm. The average aspect ratio was 1.1 in the control and the same after incubation in either fluid (Table S2). Regarding leaching, the concentrations of the investigated monomers remained in all cases below the limit of detection, which was 10 ppm for styrene and 1000 ppm for caprolactam. This is consistent with the mass balance from the lack of particle shrinkage, which indicates less than 1% of particle mass being released.

Additionally, we investigated other properties which may influence distribution and clearance of the polymer particles, which were described in detail in Santizo et al. [43]. In summary, we found that they are solid particles (measured by time-domain-NMR [59]) with no to very low surface reactivity (blank control or less than 1% of the positive control) (measured by electron paramagnetic resonance (EPR) [60]), with neutral surface charge (-2 mV) or negative surface charge (-55 mV) for PA-6 and PS-NR, respectively (measured by Zetasizer at pH 7).

### Aerosol characterization

The measured concentrations of the particles in the aerosols were close to the target concentrations (Table 5) and particle size measurements revealed an agglomeration of the nanoparticles in the inhalation atmosphere. For PA-6, cascade impactor and SMPS measurements showed that particle size increased with higher atmospheric concentrations, but particle numbers did not increase proportionally, indicating particle agglomeration. This was not seen for PS-NR, with particle size remaining constant at different concentrations and particle numbers increasing proportionally. The difference is likely due to PS-NR particles being negatively charged and repelling each other, while the neutral charge of PA-6 promotes agglomeration.

**Table 5:** Measured concentrations and particle size distributions in the generated atmospheres

Particle	Target concentration [mg/m^3^**]**	Measured atmospheric concentration		Cascade impactor measurement		SMPS measurement	
		Mean [mg/m^3^**]**	SD [mg/m^3^**]**	MMAD [µm]	GSD [µm]	TCC [N/cm^3^**]**	GMD [nm]
PS-NR	5	5.2	0.4	1.39	2.43	19,408	220
	50	50.7	2.3	1.32	2.1	139,541	253
PA-6	2	2.1	0.2	0.77	2.68	31,069	139
	10	10.3	0.6	1.24	2.20	35,325	206
	50	50.9	3.1	1.51	2.04	47,772	285

*SD* standard deviation,* MMAD* mass median aerodynamic diameter,* GSD* geometric standard deviation,* TCC* total count concentration,* GMD* geometric mean diameter. MMAD, GSD, TCC and GMD are presented as the average between four different measurements

### Clinical observations

No clinical signs of toxicity were observed, nor were there impairments in body weight development during or after the exposure. A detailed toxicology examination was conducted and will be published in a separate manuscript.

### Polystyrene detection via fluorescence

**Fig. 2:**
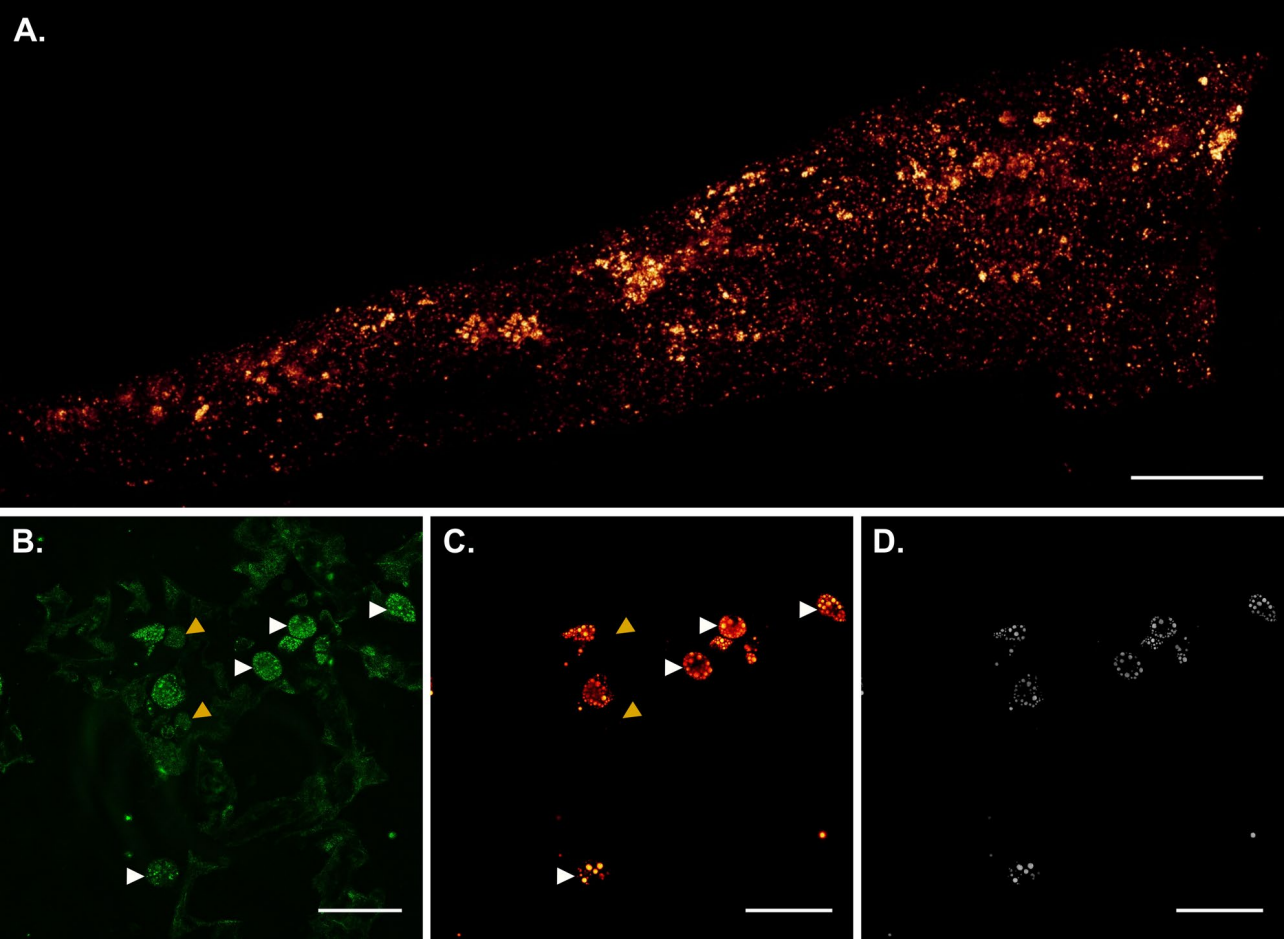
Distribution of PS-NR in lung tissue of a representative rat (50 mg/m^3^, main group). **A** Lung section taking the maximum intensity projection of stitched xyz-stacks. **B** Scattering contrast simultaneously detected with the fluorescence emission of the labeled nanoparticles in order to image the tissue structure and to identify presumed macrophages. **C** Fluorescence contrast. **D** Segmented image of **C** after processing by Labkit, ImageJ. Presumed macrophages with and without phagocytized nanoparticles are marked by white and yellow arrow heads, respectively. Scale bar is 750 μm for **A** and 50 μm for **B** to **D**. The corresponding xyz-stacks for (**B**), (**C**) and (**D**) can be found in Additional files 1, 2 and 3, respectively

In the lung, PS-NR was predominantly found as fluorescent nanoparticle agglomerates, which were distributed across the lung sections, with some areas containing a higher number of agglomerates (Fig. 2A). Differential staining of the macrophages was not successful due to the long-term storage of samples in formalin and consequent loss of epitopes. Thus, the scattering contrast was used to tentatively identify characteristic structures that were presumed to be alveolar macrophages (Fig. 2B). This analysis revealed that most nanoparticle agglomerates seemed to be wrapped inside those structures, while a few others were apparently not internalized by presumed macrophages and remained free in the lung tissue (Fig. 2C). In addition, a fraction of presumed macrophages could be identified which had not engulfed any agglomerates of nanoparticles.

**Fig. 3:**
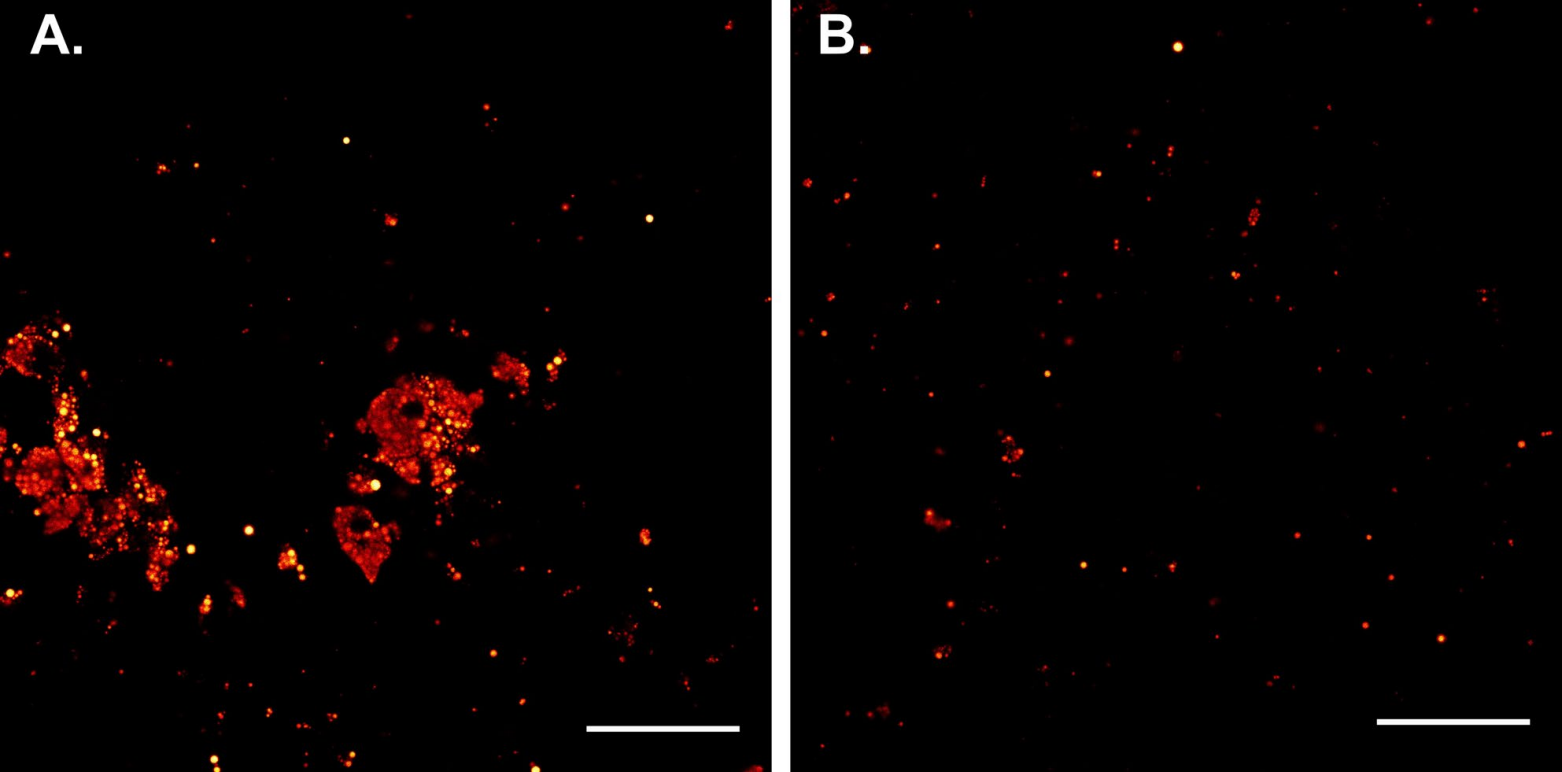
Distribution of PS-NR in the LN of a representative rat (50 mg/m^3^, main group). Representative single images of xyz stacks (Entire stacks are presented in Additional files 4 and 5) to present areas with a **A** high number of presumed macrophages, which apparently phagocytized nanoparticle agglomerates, and **B** areas with non-phagocytized nanoparticle agglomerates. Scale bar is 50 μm

In contrast to the lung, a higher number of extracellular agglomerates could be identified in the lung-draining LNs; however, densely packed agglomerates of the nanoparticles were also detected within assumed phagocytes (Fig. 3). In rats that were not exposed to nanoparticles, no fluorescent particles or agglomerates were detected.

The autofluorescence of the tissues did not compromise the detection of individual agglomerates of nanoparticles, as it was shown to be minimal in the spectral range used for the detection of Nile Red emission (Figs. 2 and 3).

**Fig. 4:**
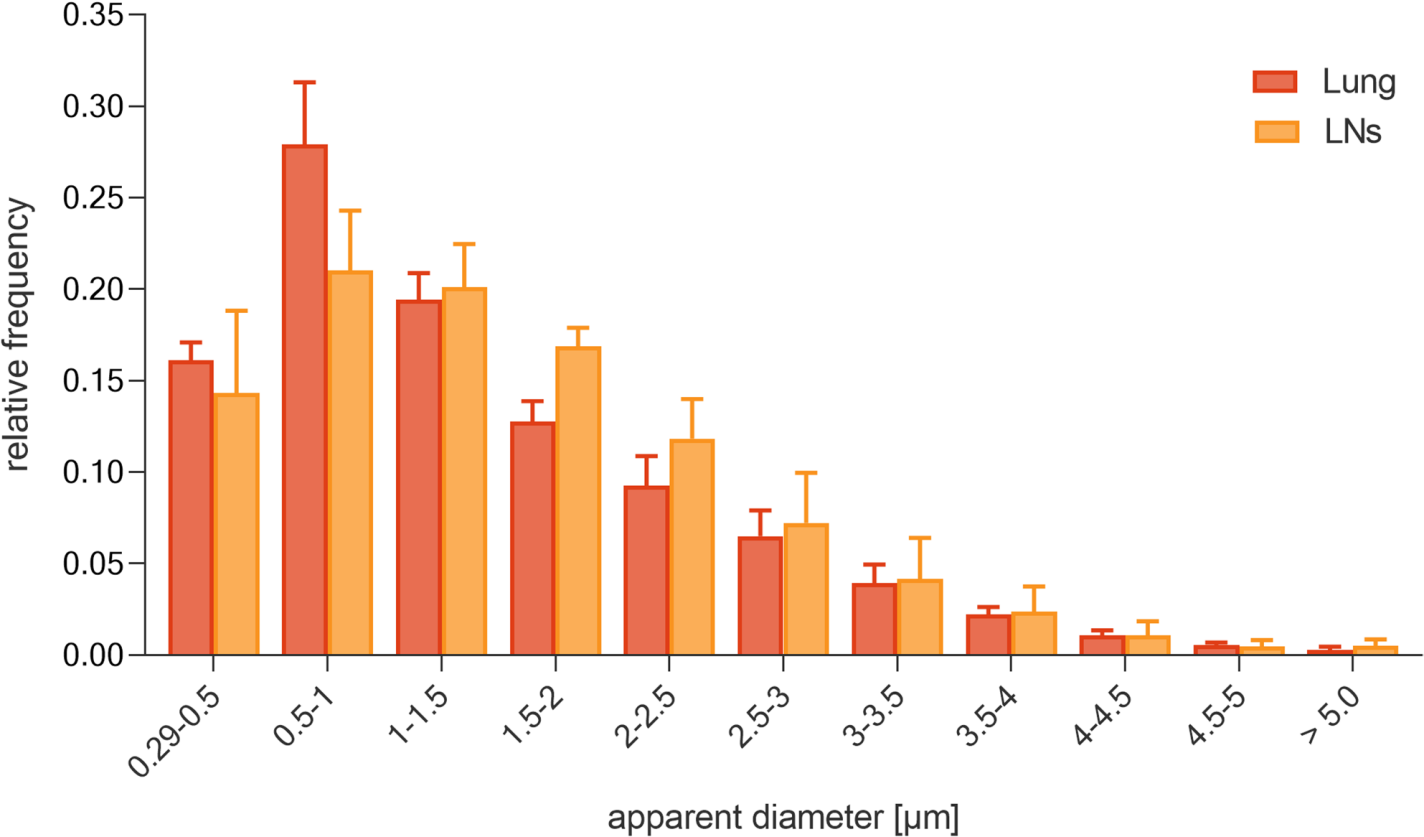
Size distribution of PS-NR particles in lung and LNs of rats (50 mg/m^3^, main group). Values are presented as mean relative frequency ± standard deviation for three animals. The apparent diameter considers the confocal imaging process of nanoparticles which appear larger than they are. Individual nanoparticles are imaged as at least 0.29 μm in equivalent sphere diameter (see Sect. "Image processing")

To quantify the size distribution of the detected fluorescent objects, the distribution of nanoparticles in all three spatial (xyz) dimensions was scanned. Fluorescent objects could be detected in the lung tissue up to an average z-depth of 53 μm for rats exposed to 50 mg/m^3^ PS-NR. The fluorescence contrast faded deeper inside the tissue, mainly due to scattering but also due to spherical aberrations, caused by refractive index mismatch of the oil-immersion objective lens and water-immersed tissue samples. Subsequently, the average intensity and the equivalent sphere diameter of each fluorescent object in 3D were determined by image segmentation based on pixel classification. The detected fluorescent objects showed a broad size distribution in both lung and LNs, with a maximum diameter of 8 μm and a maximum frequency between 0.5 and 1 μm (Fig. 4). This is reflecting sizes found in the inhalation atmosphere (Table 5). The size distribution in lung and LNs were similar.

A high inter-individual variability of number and detected mass of particles in lungs and LNs was observed (Table 6). It varied by a factor of 2 and 10 in the lungs and LNs, respectively.

An average mass of 0.42 ng of polystyrene nanoparticles was calculated for a cubic volume with an edge length of 100 μm for lung tissue. Assuming a lung volume of roughly 10 mL [61, 62], a total of 4.2 mg of PS was deposited and retained during the exposure period (2.6 mg/g lung for a mean lung weight 1.63 g ± 0.16 in the males exposed to PS 50 mg/m^3^, MG). Of this, 14.5 µg was translocated to each examined LN (assuming an approximated volume of 0.03 mL [63]) (Table 6). As several lung-draining LNs form the lymphatic system of the lung [64], the total PS mass translocated from the lungs to the LNs could sum up to approximately 100 µg.

Remote organs, namely liver, spleen and kidney, were also scanned for fluorescent nanoparticles. However, only one nanoparticle agglomerate with a diameter of 1.2 μm could be identified in kidney tissue, whereas no fluorescent objects were detected in the unexposed rats. The total volume scanned were 0.19 mm^3^, 0.1 mm^3^ and 0.14 mm^3^ for spleen, liver and kidney, respectively (Table S3). Furthermore, no fluorescent objects could be detected in blood smear of rats exposed to 50 mg/m^3^ (an area of 1.45 mm^2^ was scanned).

Additionally, rats sacrificed five weeks after the end of exposure to 50 mg/m^3^ (PEG1) were examined with respect to potential clearance of nanoparticle agglomerates from lung or LNs. Therefore, we determined again the number of fluorescent particles, their size distribution and their average fluorescence intensity for two rats of this group. The detected particle size distribution did not change significantly within five weeks of potential clearance (Figure S4). Consistent with the findings on the rats sacrificed one day after the end of exposure, the number of nanoparticle agglomerates detected varied strongly from rat to rat (Table 6), e.g., rat 31 (50 mg/m^3^ PS-NR, PEG1) exhibited a similar number of agglomerates in lung tissue as other animals exposed to the high concentration of 50 mg/m^3^ in the MG. However, only three nanoparticle agglomerates per cubic volume with an edge length of 100 μm (1 × 10^6^ µm^3^) could be spotted in its LNs. For rat 32 (50 mg/m^3^ PS-NR, PEG1), a low concentration of agglomerates was visualized in lung tissue (85 agglomerates per (100 μm)^3^) but a significantly larger number in LNs (148 agglomerates per (100 μm)^3^). Due to a slightly different particle size distribution, a similar mass uptake of PS in both tissues was observed.

**Table 6:** Absolute number and mass per (100 μm)^3^ of PS-NR particles in lung and LNs

	Animal no.	Lung		LNs	
		Absolute number of particles detected	Mass of PS per (100 μm)^3^ [ng]	Absolute number of particles detected	Mass of PS per (100 μm)^3^ [ng]
MG	26	6073	0.24	17,012	0.66
	27	9131	0.31	14,194	0.74
	28	12,261	0.72	1702	0.04
	Mean	9155	0.42	10,969	0.48
PEG1	31	10,258	0.40	14	0.01
	32	1701	0.12	3445	0.14
	Mean	5980	0.26	1730	0.07

Data represent individual animals exposed to 50 mg/m^3^ PS-NR in main group (MG) and post-exposure observation group 1 (PEG1). Values are shown as either the absolute number of particles or mass per animal, calculated according to the method described in Sect. "Image processing", Group means are also indicated. Animal numbers are described in Table S1.

The fluorescence intensity of the detected agglomerates declined slightly in lung tissue between one day and five weeks post-exposure but remained constant in the LNs (Table 7). This suggests that the particles retained their fluorescence tagging within the LNs.

**Table 7:** Fluorescence intensity of individual PS-NR agglomerates in lung and in LNs

	Lung		LNs	
	Mean [a.u.]	SD	Mean [a.u.]	SD
MG	49.7	5.5	38.3	0.6
PEG1	40	9.9	39.5	6.4

Data is shown for animals exposed to 50 mg/m^3^ PS-NR in main group (MG) and post-exposure observation group 1 (PEG1), and is expressed as mean ± standard deviation (SD) for three animals in MG and two animals in PEG1. A.u. = arbitrary units

### Polyamide detection and quantification with Py-GC/MS

**Fig. 5:**
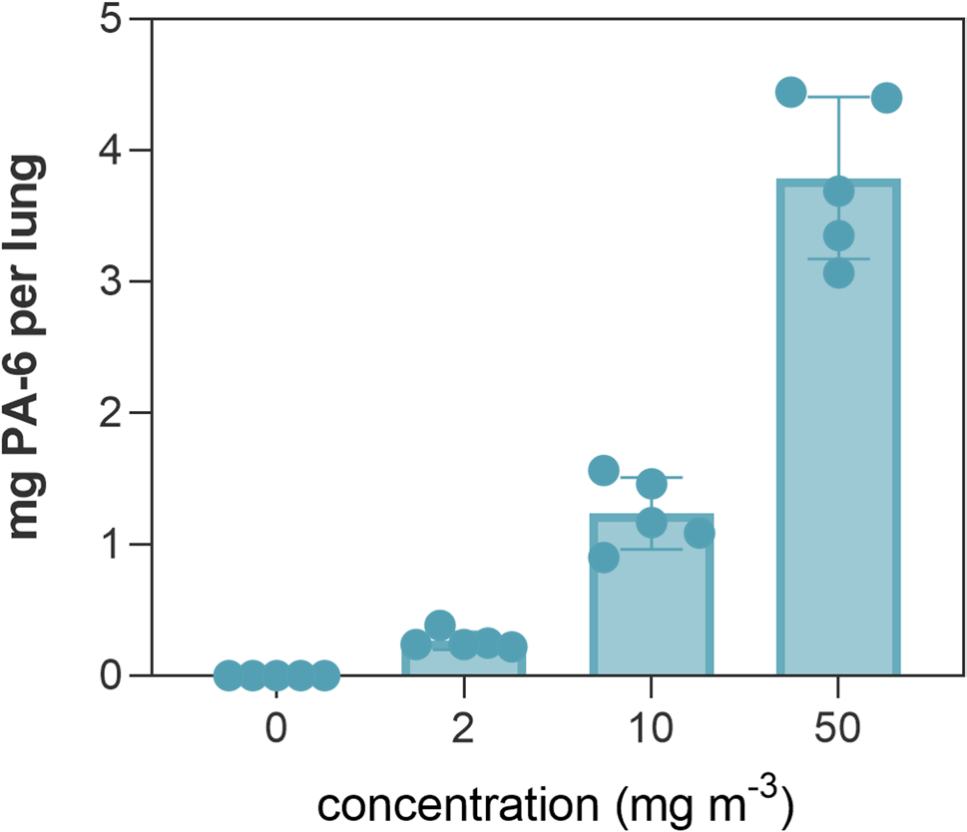
Total mass of PA-6 per lung of rats from the main group. The total amount to PA-6 is calculated based on the wet weight of lungs. The data are expressed as mean ± standard deviation for five animals per group

Py-GC/MS showed an increase of PA-6 in the lungs with higher aerosol concentrations (Fig. 5), reaching an average of 0.26, 1.2 and 3.8 mg per lung (organ wet weight) at the atmosphere concentrations of 2, 10 and 50 mg/m^3^, respectively. This corresponds to 0.3, 1.3, 3.6 mg PA-6/g lung (for mean lung weights of 0.91 g ± 0.07, 0.94 g ± 0.04, 1.05 g ± 0.13, respectively).

**Fig. 6:**
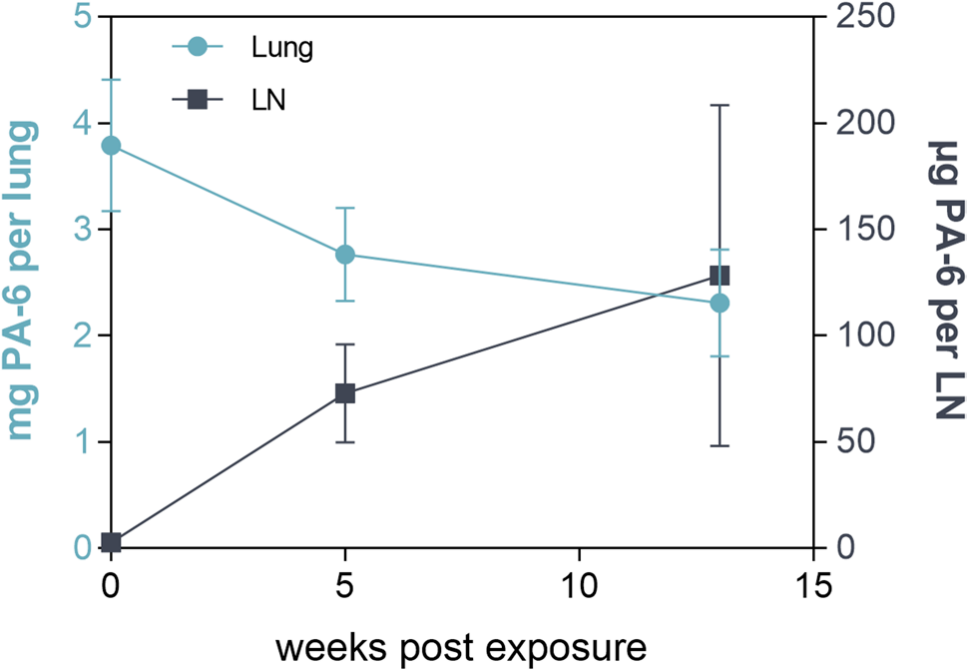
Total mass of PA-6 per lung and per LN of rats during recovery periods. Data are shown for animals exposed to 50 mg/m^3^ PA-6. The total amount of PA-6 was calculated based on the wet weight of lungs and LNs. Results are expressed as mean ± standard deviation for five animals per group, except for LNs in PEG1, which includes only four animals

The lung burdens decreased after the end of the exposure. After 13 weeks, an average of 2.31 mg of PA-6 was still present in the lung. At the same time, PA-6 in the LNs increased after the end of the exposure, from a mean burden of 2.95 µg per LN to 126.29 µg per LN (Fig. 6). In one animal from PEG1, PA-6 burdens in the LN were found to be below the detection limit at five weeks after the end of the exposure (data not shown).

Merely, in one animal exposed to 10 mg/m^3^ PA-6, the mass in the LN was above the limit of detection: 1.34 µg of PA-6 in 6.47 mg of LN (organ wet weight). In the LNs of all animals exposed to 2 mg/m^3^, PA-6 was below the limit of detection (1.2 µg).

### Summary of study and findings

In Table 8, study characteristics and results obtained for each particle are summarized.

**Table 8:** Summary of study characteristics and findings for each particle

	PS-NR	PA-6
Particles		
Mean particle size (d50 in mass metrics) [nm]	89	66.7
MMAD [µm]	1.32	1.51
In vivo experiment		
Sex	Male	Female
Mean weight [g]	270	200
Calculated ventilation rate [L/min/kg b.w.]	0.22	0.16
Concentrations evaluated [mg/m^3^]	50	2, 10 and 50
Total inhaled mass for 50 mg/m^3^ [mg]	77.8	57.6
Timepoints evaluated [weeks post exposure]	0 and 5	0, 5 and 13
Detection method	Confocal microscopy	Pyrolysis-GC/MS
Limit of detection (LOD) / Limit of quantification (LOQ)^a^	9.3E-13 µm^3^	LOD = 1.2 µg/LN LOQ = 1.5 µg/LN LOD = 36 µg/lung LOQ = 100 µg/lung
Results at the end of the exposure to 50 mg/m^3^		
Detected mass		
Lung [mg/lung]	4.2	3.8
LNs [µg/LN]	14.5	2.95
Deposition [% total inhaled mass]	5.4	6.6
Retention half-time [days]	46	68 (initial phase) and 226 (slow phase)

^a^ LOD = LOQ for PS. The LOQ is defined as one agglomerate with a volume of 0.0004 µm^3^ within an examined volume of 0.43 mm^3^. In the case of PA-6, LOQ was calculated based on the lowest value in calibration and its standard deviation and respective dilution factor for the organs

## Discussion

Nanoplastic particles were quantified in the lungs and lung-draining LNs of rats exposed via inhalation to two different nanoplastics (PS and PA-6) at different exposure concentrations and at various timepoints. These data improve our understanding of how nanoplastics are deposited in and distributed from the lung. It also calls attention to the challenges of obtaining such data: starting from sample preparation and removal of biological matrix to the actual quantification of nanoplastics in biological matrices. Each step will be discussed in detail in the following sections.

### Labeling of PS particles

Fluorescent labeling is a widely used method to facilitate the tracking of particles within organisms due to its cost-effectiveness, safety, and high sensitivity; however, it also presents limitations, including autofluorescence of the tissue, the potential dye leakage or loss of fluorescence over time [65, 66]. Therefore, prior to the study, the suitability of different dyes was investigated.

To avoid issues with tissue autofluorescence and enable sensitive fluorescence detection, it is advantageous to stain the polymer nanoparticles with fluorophores emitting in the red region of the spectrum, since tissues exhibit autofluorescence predominantly in the ultraviolet and blue regions of spectrum [67]. Fluorescein, for example, falls within the range of the autofluorescence of the tissue, hence being considered unsuitable. In contrast, Nile Red and Nile Blue can be used to label polymeric materials [68–70] and their emission falls within the suitable spectral range. Nile Blue can be covalently bound to the PS chain during the polymerization process, which minimizes leakage. However, only a limited amount of the dye was bound into the polymer molecule, resulting in a low sensitivity for quantification (data not shown). On the other hand, Nile Red was incorporated by a swelling method [39], and yielded a higher fluorescence intensity per particle than Nile Blue. The stability of the Nile Red labeling was investigated in a nonpolar acrylate matrix for five days, and no leaching could be detected (Figure S5). Taken together, Nile Red was chosen as most appropriate fluorescent probe within our study.

### Challenges in sample preparation

Micro- and nanoplastic quantification usually requires separation from the tissue. This should remove the biological matrix while not damaging the targeted plastic particles. Different extraction methods can yield varying recoveries of the polymers [32].

Prior to the in vivo study, the suitability of different methodologies for separation of the particles from the tissue were tested. It was crucial to maintain the Nile Red dye within the PS-NR particles as it was not covalently bound to the polymer chain. Chemical digestion using basic piranha and Soluene^®^ 350, and chemical extraction using tetrahydrofurane (THF) caused either excessive foam formation, degraded the particles or simply failed to retrieve the particles. Enzymatic digestion using protease and surfactants yielded a low recovery of PA-6 and caused leaching of the Nile Red dye from PS. This low recovery may result from a destruction of the PA-6 polymers or, more likely, the subsequent ultracentrifugation, did not effectively form a PA-6 pellet because of the low density of particle aggregates. Similar challenges with extracting polymers from biological matrices have been widely reported in literature, with particle damage and incomplete recovery being the main issues [32, 33, 71, 72].

The quantification of PA-6 in liver tissue presented another challenge. The extraction of PA-6 using HFIP from lungs and LNs allowed quantifications in the mg/kg concentration range with good recovery. In the liver, however, excessive contamination by matrix pyrolysis products in the GC/MS instrument inlet prevented PA-6 quantification in the liver. SPE was tested as an alternative method. As shown in Table S4, a significant background contamination of PA-6 was observed in three out of 11 tested SPE cartridges with different types of solid phase material and from different manufacturers. Alternatively, sample work-up by preparative ultracentrifugation (PUC) or by SPE with protein capture and/or ionic stationary phases could be promising approaches.

Ultimately, PS-NR particles were not separated from the tissue, but were quantified in lung, LNs, liver, kidney and spleen by confocal laser microscopy with a semi-automatic imaging software, and PA-6 was extracted using HFIP and quantified by Py-GC/MS in the lung and LNs.

### Lung burden and biopersistence

The inhalation of aerosols containing 50 mg/m^3^ of nanoplastics particles yielded lung burden of 4.2 mg for PS (2.6 mg/g lung) and 3.8 mg for PA-6 (3.6 mg/g lung) after 28 days. This corresponds to 5.4% and 6.6% of the total inhaled mass of approximately 77.8 mg for PS (ventilation rate of 0.22 L/min for male rats) and 57.6 mg for PA-6 (ventilation rate of 0.16 L/min for female rats), respectively. For comparison, we employed the MPPD model to calculate the expected lung deposition. The predicted deposition for PS was 6.8% and for PA-6 was 6.9% in the tracheobronchial and alveolar regions, being in the same order of magnitude of the quantified values (Figure S6).

Morrow [73] proposed, after a careful analysis of published data, that when the exposure rates to poorly soluble low-toxicity particles exceed the clearance capacity of the lungs, alveolar macrophages are impaired, leading to the induction of inflammatory responses and an increase in the retention time. This condition was named “lung particle overload”, and the threshold for the initiation of these effects was then defined as 1 mg/g lung for a particle with unite density of 1 g/mL.

Physical-chemical characterization of both polymers revealed no surface reactivity and no solubility. Accordingly, PS and PA-6 can be considered poorly soluble particles with potentially low toxicity. The measured lung burdens for PS at 50 mg/m^3^ (2.6 mg/g lung) and PA-6 at 10 (1.3 mg/g lung) and 50 mg/m^3^ (3.6 mg/g lung) exceeded Morrow’s lung threshold for the initiation of lung overload effects. Thus, the data indicate that lung overload occurred for both polymers at least at 50 mg/m^3^.

In addition, under lung overload conditions, the retention time in the lungs is increased. Lung clearance of poorly soluble particles can be treated as first-order kinetics reaction [74]; thus, an estimated retention half-time can be calculated to evaluate the biopersistence of a particle in the lungs [56, 73, 75]. For PS-NR, the retention half-time could only be calculated up to five weeks post-exposure, resulting in an estimated retention half-time of 46 days. In a related study, Sarlo [76] reported retention half-times of 53.4, 43.4 and 38.8 days for PS particles of three different sizes (20, 100, and 1000 nm, respectively) after a total of ten repeated pharyngeal aspiration exposures. Our calculated retention half-time value was within those published by Sarlo et al. In both studies, the retention half-times are close to the physiological half-time of 60 to 80 days for poorly soluble particles in rats [77]. However, because only two PS timepoints were measured, we cannot determine if retention time slowed after five weeks of recovery. Although our estimated retention half-time aligns with previous studies, the small sample size and limited timepoints reduce the accuracy of this value.

For PA-6, the data revealed a two-phase clearance pattern. The initial phase, occurring up to five weeks post-exposure, is characterized by a rapid clearance with a calculated retention half-time of 68 days. This phase was also within the physiological half-time for poorly soluble particles After this, from five to 13 weeks post-exposure, particles are cleared slower with a half-time of 226 days. Such two-phase clearance is commonly observed with particles. In the early, fast phase, the particles deposited in the tracheobronchial region are primarily cleared from the lung via the mucociliary escalator, while the later, slow phase, represents particle clearance from the alveolar region [78, 79]. As the lung overload refers to the cessation in the alveolar macrophage clearance, the slow phase clearance observed (226 days) is another indication of the occurrence of lung overload. However, a definitive conclusion about lung overload would require data from more concentrations in the recovery groups.

Interestingly, Warheit et al. [80] demonstrated the opposite clearance pattern for nylon-6,6 fibers in male rats subjected to the same exposure regimen as in our study (6 h per day, five days per week, over four weeks), albeit at a lower concentration (19.6 mg/m^3^). A slow clearance phase was observed until the first month, whereas a rapid clearance phase was demonstrated between one and three months. Three months after the end of the exposure, less than 20% of the initial burden was in the lungs. In contrast, around 60% of the initial PA-6 burden remained in the lungs three months (13 weeks) after the end of the exposure in our study. These differences are most likely linked to different lung burdens. In our study, the high concentration of 50 mg/m^3^ resulted in a lung burden that exceeded the established lung overload threshold. It is worth noting that our investigation did not include assessments of lung burden following the recovery period for the mid and low dose concentrations, which could present a gap in our findings, as lower concentrations are generally expected to result in burdens that do not reach the critical overload threshold. The concentrations administered by Warheit et al. [80] were probably below the lung overload threshold, with the highest concentration of 19.6 mg/m^3^. Another reason for the difference in clearance rate may relate to the method of quantification. The images presented in the Warheit paper revealed that the sample contained not only fibers but also a considerable fraction of small particles. The lung burden was determined by counting exclusively the number of fibers (> 5 μm) using phase-contrast light microscopy after appropriate digestion and filtration. Using this method, smaller particles were evidently not considered in the lung burden, resulting in a clearance rate specific only to fibers, which might differ from particles. Therefore, this result cannot be directly compared with our findings.

PS was apparently cleared from the lungs more rapidly than PA-6 under similar conditions. Despite the limitations related to the quantification method used for PS, our findings align with existing literature, which supports the validity of our data. However, definitive conclusions cannot be drawn based on this data only, because lung burdens of PS were measured only at two timepoints in a few animals. Moreover, the confocal laser microscopic method is a semi-quantitative method, in contrast to pyrolysis GC used for analyzing PA-6.

### Bioavailability and distribution of polymer particles upon inhalation exposure

We were able to demonstrate that PS and PA-6 particles deposit in the lungs and translocate to the lung-draining LNs. The translocation of inhaled particles to the lung-draining LNs has been extensively documented in the literature, as reviewed by Nakane [81]. This translocation can occur via cells (e.g., by alveolar macrophages) [82] or extracellularly, in which free particles move to the interstitium and are transported with the lymphatic fluid to the LNs [83]. We have observed the presence of PS-NR both within presumed macrophages and as free particles in the LNs. It is, however, unclear whether both translocation mechanisms occurred, if particles were released following macrophage cell death in the LNs, or if particles were phagocytized in the LNs by resident phagocytes.

PA-6 particles also translocated from the lungs to the lung-draining LNs. The detected amounts increased in the LNs and decreased in the lungs over time after the end of the exposure. This demonstrates the particle clearance from the lung via the interstitium and lymph vessels. Several studies (epidemiological [84–87] and experimental in vivo [49, 80, 87, 88]) investigated PA’s respiratory toxicity. In contrast, to our knowledge, this is the only study which investigated the toxicokinetics of PA-6 particles; while Warheit et al. [80] has addressed PA-6,6 fibers and exclusively in the lungs, without providing information on LNs.

For PS-NR nanoplastic particles, the translocation to extrapulmonary organs and in blood was investigated. Except for one particle agglomerate identified in one kidney of one animal, no particles were detected in any of the investigated organs or the blood. In a study with repeated pharyngeal aspiration of PS particles in rats, Sarlo et al. [76] reported that most of the 100 nm and 1000 nm particles remained in the lungs up to four months with minimal translocation to remote organs (< 10% total detected particles), while more translocation was observed with 20 nm particles. Delaney, Rodriguez [89] reported that the majority of both 20 nm and 1 μm PS particles remained in the lungs up to a week after single intratracheal instillation, with the smaller particles showing greater retention compared to their larger counterparts, opposite to the size related tendency observed by Sarlo et al. By contrast, several studies have demonstrated the translocation of PS to extrapulmonary organs, including brain, liver, kidney, spleen, and placenta [90–94]. It should be noted that the PS particles used in the different studies vary by their physicochemical properties, and previous studies predominantly utilized mice instead of rats. The fact that our findings do not indicate translocation of PS to extrapulmonary organs, while different studies reported such translocation, might be attributed to different detection methods. Most studies used qualitative techniques such as whole-body or whole organ measurements of fluorescence or radioactivity, whereas we measured particles in tissue sections. As a result, the limit of detection could be lower in our study. In any case, these findings suggest that the translocation of particles to remote organs is minimal. Further investigations are needed to accurately quantify translocation and establish potential differences of different PS particles.

### Limitations of the study

The quantification of fluorescence via confocal microscopy is limited by the volume of tissue scanned and assumes that the particle deposition occurs evenly throughout the lung after inhalation [95]. Additionally, although this method is semi-automated, it is still time-consuming, which limits the number of regions per sample and the total samples that can be examined. Thus, at the current stage, it is rather semi-quantitative, and quantified values should be interpreted with caution. Still, the lung burden determined by this method for PS at the concentration of 50 mg/m³ was in the same order of magnitude of the one predicted by the MPPD model; it was also comparable to the lung burden of PA-6 (50 mg/m³), which was quantified by Pyrolysis-GC/MS.

The PS test material of the present study was synthesized by emulsion polymerization, and thus it shares some commonalities with the much criticized PS beads, such as the spherical shape [40]. However, we mitigated the known issue of surfactant and oligomer content by washing the PS-NR three times in water and then added a minimal content of surfactant to ensure stability before dosing. No biocide was employed. In addition, the fluorescent dye, Nile Red, was not covalently bound to the PS and hence could leak from the particles. There were, however, no signs of leakage when the particles were embedded in a nonpolar matrix and no background or unspecific staining in the tissue sections were observed. A minor fluorescence decline was observed in nanoplastics in lung tissue, 20% over five weeks.

Confocal laser microscopy could detect a single PS-NR particle agglomerate with a volume of approximately 0.0004 µm^3^ in a scanned tissue volume of approximately 0.43 mm^3^. No particles were found in control tissues. To further enhance sensitivity, scanning larger volumes, employing light sheet microscopy and using tissue clearing techniques could be employed [96, 97].

Pyrolysis-GC/MS proved to be a sensitive, specific and reproducible method for the detection and quantification of PA-6 in the lung and the LNs. In liver tissue, however, the matrix could not be sufficiently removed and caused high background levels; thus, the method was unfeasible for PA-6 quantification in this organ (see 4.2). Appropriate methods for the digestion and particle isolation from the lipid-rich and proteinaceous matrices are still needed.

We are aware that most PA microplastics derive from textiles. However, previous studies have shown that a large proportion of PA microplastics in indoor air, especially the smaller fraction, are indeed non-fibrous fragments [34–36]. In fact, in human lung tissues derived from autopsies, PA was mostly detected as particles instead of fibers [37]. One should finally note that the specific PA-6 particles were realistic in their non-spherical shape, but contained a small amount of PVA to prevent them from aggregation [43]. Although PVA was not covalently bound to the PA-6, its presence cannot be excluded as a factor affecting the biokinetics.

Although different study designs were employed for testing PS and PA-6, the primary goal of this study was not to compare the toxicokinetic profiles of different polymers; instead, we aimed to evaluate methodologies for detecting and quantifying nanoplastics in biological tissues and to provide realistic insights into their toxicokinetics while acknowledging the limitations of the proposed methods.

## Conclusion

Our study evaluated two different approaches for quantifying two different nanoplastics particles in tissues. Confocal fluorescence microscopy demonstrated a high potential for becoming a reliable method for detecting and quantifying fluorescent-labeled nanoplastics. Unlike many other techniques, it does not require sample preparation to remove biological matrix, which is a major challenge in quantifying micro- and nanoplastics. However, its current limitation lies in the relatively small tissue volumes and limited number of samples that can be examined. Future advances in automated, high-throughput scanning technologies could enable the analysis of larger tissue volumes and more samples, thereby increasing the method’s power and applicability.

PA-6 in lung and LNs was successfully quantified by Py-GC/MS following solvent extraction from the tissue matrix, with detection achieved in the lungs even at the low exposure concentration of 2 mg/m^3^. This was, however, not feasible in the liver and other organs. More efforts need to be made in sample preparation process to reliably remove confounding matrix, especially in fat-rich tissues like liver.

With the feasible quantification methods, we could obtain data on the toxicokinetics of inhaled nanoplastic particles: the lung burdens and clearance and distribution from the lung into other tissues. PS was found in the lung and its draining LNs. There was one particle agglomerate detected in a kidney, but otherwise no distribution to extrapulmonary organs or in the blood was observed – within the detection limits of the analytical method. Inhaled PA-6 deposits in the lung and slowly distributes from there into the draining LNs. 5.4 and 6.6% of the inhaled PS-NR and PA-6, respectively, were deposited in the lungs. For each of the tested particles, inhalation of 50 mg/m^3^ for 28 days yielded lung burdens above lung overload threshold of 1 mg/g lung. After the end of the exposure, PS particles were cleared from the lung with a half-time of 46 days (calculated up to five weeks post-exposure) and PA-6 particles were cleared with an initial half time of 68 days (up to five weeks post-exposure) and a long-term half-time of 226 days (after five weeks up to 13 weeks post-exposure).

In summary, our study proposes methods to address current gaps in nanoplastic detection in biological tissue and offers new perspectives on polymer toxicokinetics that will support ongoing research and risk assessment of nanoplastics.

## Supplementary Information

Below is the link to the electronic supplementary material.

Supplementary Material 1

Supplementary Material 2

Supplementary Material 3

Supplementary Material 4

Supplementary Material 5

Supplementary Material 6

## Data Availability

The datasets used and/or analyzed during the current study are available from the corresponding author on reasonable request.
