# Supplementary material for: Lung retention, distribution and persistence of polymer particles in rats exposed via inhalation
Source: Part Fibre Toxicol. 2026 Jan 17;23:2. doi: 10.1186/s12989-025-00655-4 (PMC12814592; doi:10.1186/s12989-025-00655-4)
Supplement: Supplementary file 6 — Supplementary Material 6 [file 12989_2025_655_MOESM6_ESM.docx]

**Supplementary information**

**Supplementary information on polystyrene synthesis and labelling**

**Synthesis of Polystyrene-Nile Blue dispersion**

For the synthesis of covalently labelled polystyrene, three different concentrations of Nile Blue methacrylamide (NBM) were incorporated to the styrene emulsion: 0.0375, 0.075 and 0.150 parts per hundred parts of monomer (pphm). The remainder of the procedure followed the same protocol as for the unlabeled polystyrene dispersion, as detailed in section 2.2.

**Fluorescence properties**

The incorporation of the NBM was evaluated by separating the serum from the labelled PS dispersion by ultracentrifugation (40000 rpm for 3 h). The fluorescence intensity (excitation at 618 nm, emission at 680 nm) of the diluted polymer dispersions (1:250) was a factor of 500 – 1000 higher than that of the equivalently diluted serum. Therefore, it can be assumed that most of the dye was incorporated into the particles.

Nevertheless, it was observed by fluorescence microscopy that the particles with a NBM concentration of 0.150 pphm exhibited a factor of 10 lower fluorescence intensity than the particles labelled with Nile Red via the swelling method.

**Supplementary Figures**


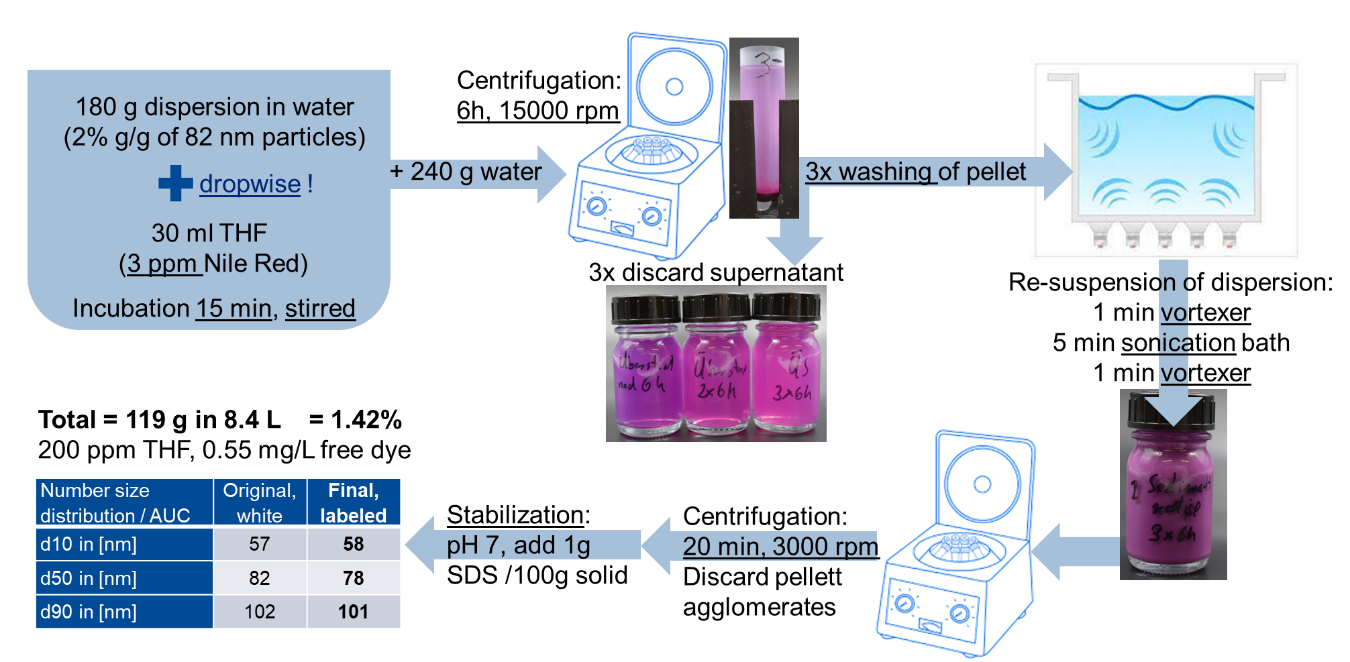


**Figure S1.** Summary of the labelling protocol for PS-NR.

**A.**

**B.**

**Figure S2.** Size distribution of (A) PS-NR and (B) PA-6 in suspension by AUC in mass metrics with TEM evaluation (n > 1000 particles).


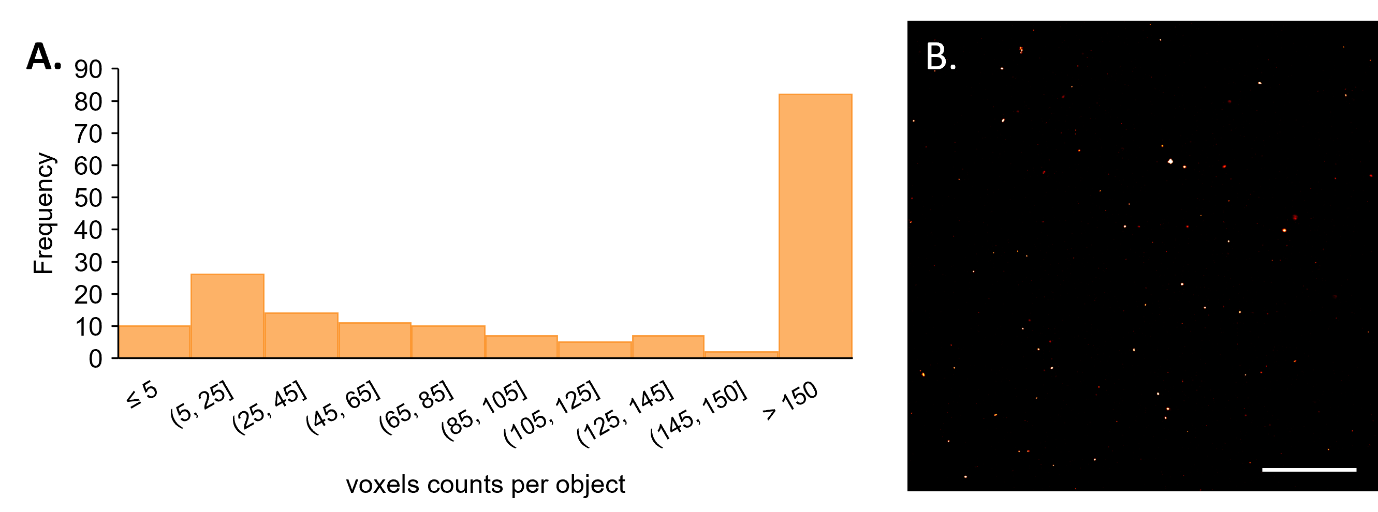


**Figure S3.** PS-NR nanoparticles embedded in a dried Acronal 3659X film (1:500). (A) Voxel counts of each detected object by segmentation procedure (the same as for the tissue samples) but recorded under the more sensitive imaging settings (4x averaging, etc.). (B) Single section of an image stack which shows the distribution of nanoparticles in the Acronal film. Scale bar 50 µm.


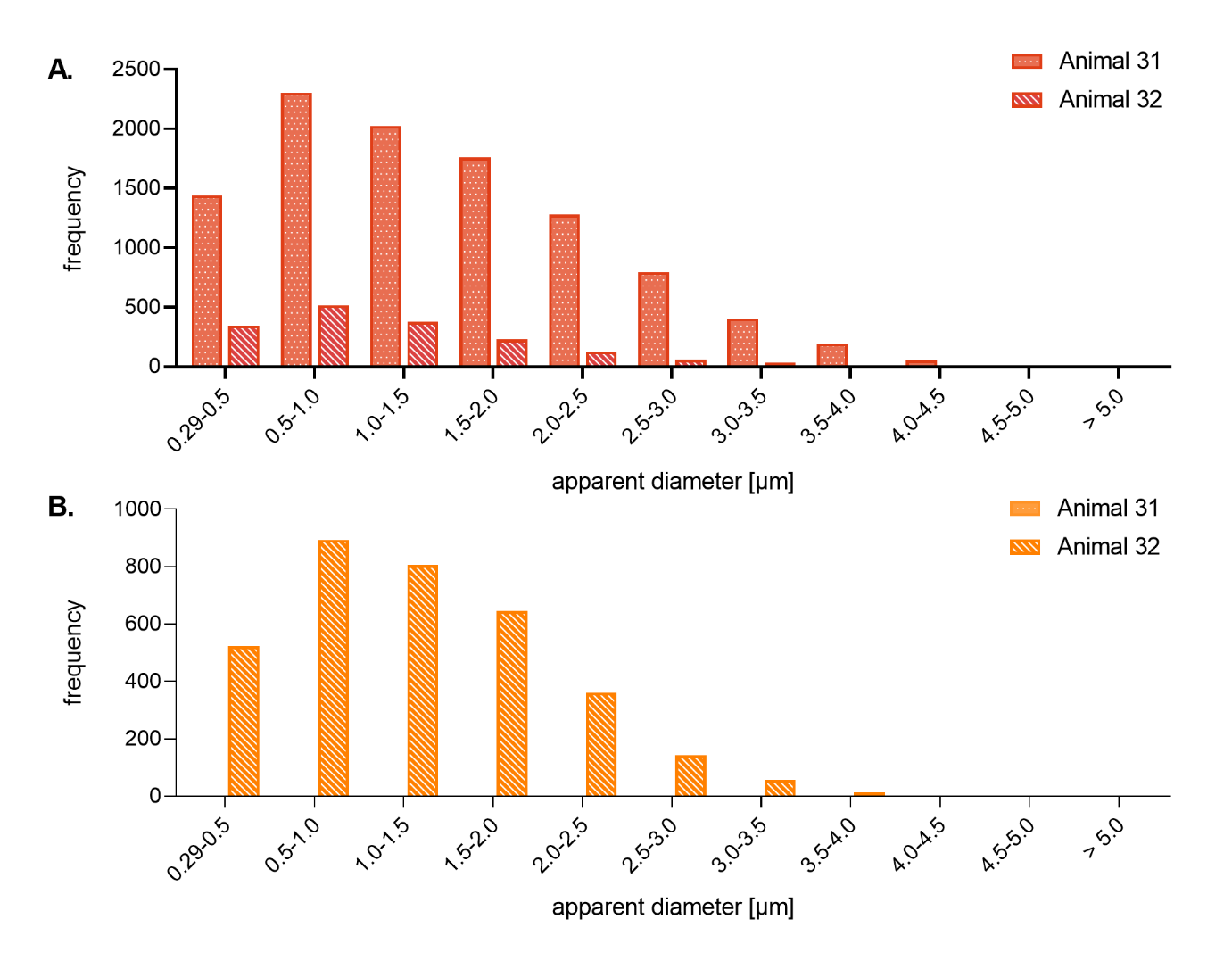


**Figure S4**. Absolute particle size distribution of PS-NR particles in post-exposure observation group 1. Results are shown for (A) lung and (B) lymph nodes (LNs) of two animals from PEG1 exposed to 50 mg/m³ PS-NR. In LN of animal 31, the absolute number of particles detected per bin was consistently ≤ 4.


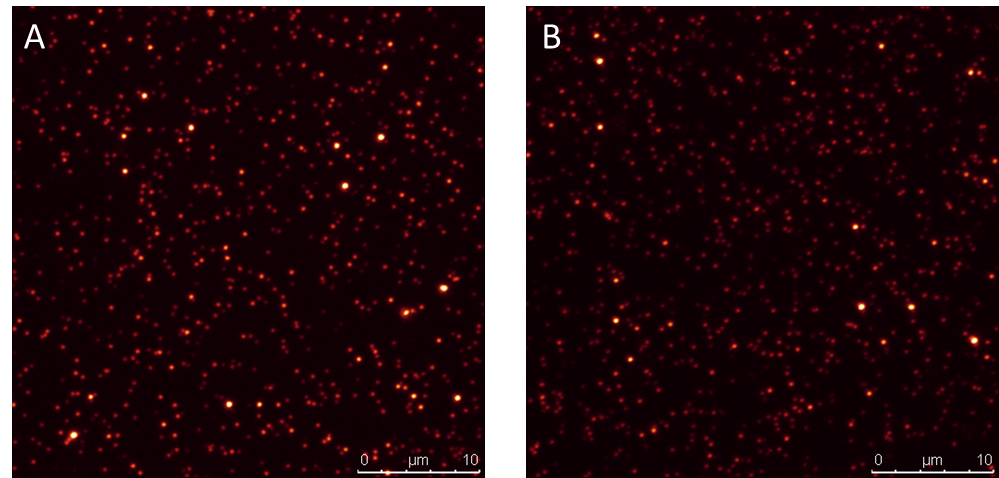


**Figure S5**. PS-NR particles embedded in Acronal 3659X (1:1000). (A) directly after drying at 70°C and (B) 5 days later. Maximum projections of xyz-stacks with a depth of 10 µm


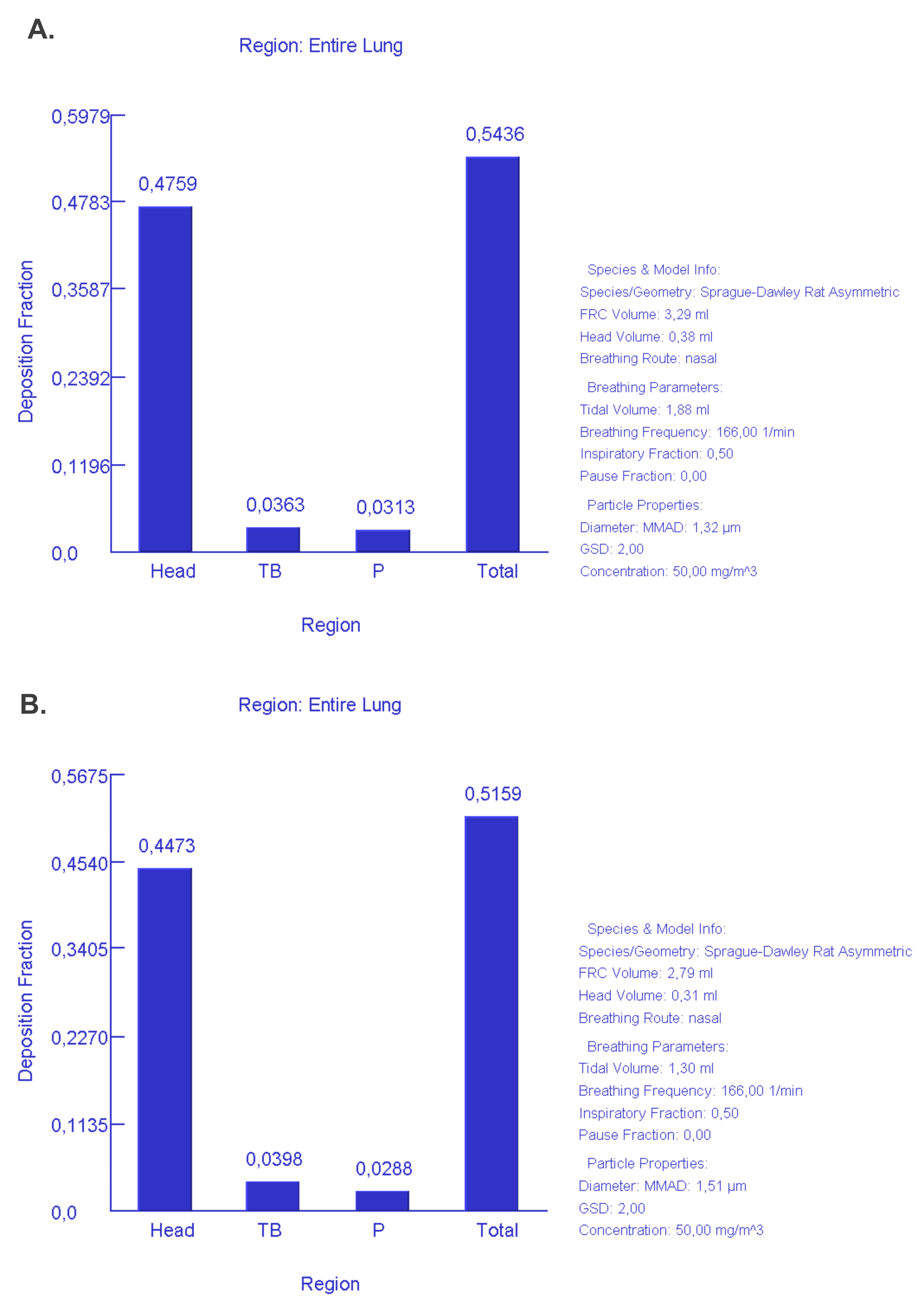


**Figure S6.** Deposition fractions predicted using MPPD model for PS-NR (A) and PA-6 (B) using parameters described in Table 4. The fractions considered for our study were the sum between tracheobronchial (TB) and pulmonary (P) fractions.

**Supplementary Tables**

**Table S1.** Detailed description of test groups in the in vivo inhalation study.

|  | **Concentrations** | **Post exposure** | | **Animal numbers** |
| --- | --- | --- | --- | --- |
|  | **[mg/m^3^]** | **[weeks]** | **[days]** |  |
|  | ***PS-NR*** | | | |
| *Main Group (MG)* | 0 | 0 | 1 | 1 - 5 |
|  | 5 |  |  | 16 - 20 |
|  | 50 |  |  | 26 - 30 |
| *Post-exposure group 1 (PEG1)* | 0 | 5 | 33 | 6 - 10 |
|  | 5 |  |  | 21 - 25 |
|  | 50 |  |  | 31 - 35 |
| *Post-exposure group 2 (PEG2)* | 0 | 13 | 91 | 11 - 15 |
|  | 50 |  |  | 36 - 40 |
|  | ***PA-6*** | | | |
| *Main Group (MG)* | 0 | 0 | 1 | 51 - 55 |
|  | 2 |  |  | 66 - 70 |
|  | 10 |  |  | 71 - 75 |
|  | 50 |  |  | 76 - 80 |
| *Post-exposure group 1 (PEG1)* | 0 | 5 | 32 | 56 - 60 |
|  | 50 |  |  | 81 - 85 |
| *Post-exposure group 2 (PEG2)* | 0 | 13 | 90 | 61 - 65 |
|  | 50 |  |  | 86 - 60 |

**Table S2.** Results of the testing for PS-NR and PA-6 stability against dissolution or transformation.

| **Polymer particle** | **Dissolution condition** | **Median size  (D50)**  **[nm]** | **Average size**  **± SD**  **[nm]** | **Median aspect ratio**  **(rel. number of particles > 3:1)** |
| --- | --- | --- | --- | --- |
| *PS-NR* | Reference in H_2_O | 75.7 | 74.3 ± 16.3 | 1.1 (0%) |
|  | Gamble pH 7.4 | 76.0 | 75.6 ± 15.2 | 1.1 (0%) |
|  | PSF pH 4.5 | 75.2 | 74.0 ± 17.2 | 1.1 (0%) |
| *PA-6* | Reference in H_2_O | 49.0 | 60.4 ± 39.8 | 1.5 (5.5%) |
|  | Gamble pH 7.4 | 49.1 | 62.8 ± 45.0 | 1.6 (6.5%) |
|  | PSF pH 4.5 | 50.0 | 63.7 ± 46.5 | 1.6 (5.1%) |

Testing was performed with a continuous flow system (CFS) with TEM evaluation (n > 1000 particles).

**Table S3.** Scanned regions, volumes or area, and number of detected PS-NR particles in remote tissues.

| **tissue**​ | ***without averaging ​*** | | ***with averaging*** | | **total scanned volume  [mm^3^]**​ | **scanned area**​ **[mm^2^]​** | **number of detected particles**​ |
| --- | --- | --- | --- | --- | --- | --- | --- |
|  | ***(lower sensitivity)​*** | | ***(higher sensitivity)​*** | |  |  |  |
|  | **number of regions scanned**​ | **effective scanned volume  [mm^3^]**​ | **number of regions scanned**​ | **effective scanned volume  [mm^3^]**​ |  |  |  |
| *spleen​* | 20 (26),15 (27),15 (28)​ | 0.18​ | 13 (26)​ | 0.006​ | 0.19​ |  | 0​ |
| *liver​* | 20 (27)​ | 0.07​ | 20 (26), 20 (28)​ | 0.03​ | 0.1​ |  | 0​ |
| *kidney​* | 22 (26)​ | 0.08​ | 20 (27), 20 (28), 10 (31)​ | 0.025 (27-28) + 0.033 (31) = 0.058​ | 0.14​ |  | 1 (31)​ |
| *blood​* | ​ | ​ | 10 (27), 10 (28), 4 (29)​ |  | ​ | 1.45 | 0 |

Data is shown for spleen, liver, kidney, and blood of animals exposed to 50 mg/m^3^ PS-NR in main group (26, 27 and 28) and post-exposure observation group 1 (31 and 32). The animal numbers corresponding to the specific number of scanned regions and/or detected particles are indicated in parentheses.

**Table S4.** Overview of PA-6 background observed in the HFIP extract from different types of solid phase material and different manufacturers.

| **SPE cartridge ​(manufact.)​** | **rinse volume​** | **mass of PA6​** | **mass of PA6​** |
| --- | --- | --- | --- |
|  |  | **in 100µl​** | **per cartridge​** |
|  | **[ml]​** | **[ng]​** | **[µg]​** |
| *C18 G (MN)​* | ~2​ | 1220​ | 24.4​ |
| *C18 (MN)​* | ~2​ | 400​ | 8.0​ |
| *SiOH (MN)​* | ~2​ | 25​ | 0.5​ |
| *Hypercarb (Thermo)​* | ~2​ | 0​ | 0​ |
| *Hypersep Si (Thermo)​* | 1.7​ | 0​ | 0​ |
| *Leer + Sand (MN)​* | 1.7​ | 0**​ | 0**​ |
| *CN modified (MN)​* | 1.7​ | 0**​ | 0**​ |
| *ODS-AQ (YMC) *​* | 1.7​ | 0**​ | 0**​ |
| *C18-EW (Agilent)​* | 1.7​ | 0**​ | 0**​ |
| *Diol (Agilent)​* | 1.7​ | 0**​ | 0**​ |
| *CN (Agilent)​* | 1.7​ | 0**​ | 0**​ |

** Mass spectrometry trace visible below limit of quantification.
